# The ongoing impacts of hepatitis c - a systematic narrative review of the literature

**DOI:** 10.1186/1471-2458-12-672

**Published:** 2012-08-18

**Authors:** Emma R Miller, Stephen McNally, Jack Wallace, Marisa Schlichthorst

**Affiliations:** 1 Discipline of General Practice, School of Population Health, University of Adelaide, Adelaide, 5005, South Australia; 2 Australian Research Centre in Sex, Health & Society, La Trobe University, Melbourne, Australia; 3 School of Population Health, University of Melbourne, Melbourne, Australia

**Keywords:** Hepatitis C, Health outcome, Social impact

## Abstract

**Background:**

Many countries have developed, or are developing, national strategies aimed at reducing the harms associated with hepatitis C infection. Making these strategies relevant to the vast majority of those affected by hepatitis C requires a more complete understanding of the short and longer term impacts of infection. We used a systematic approach to scope the literature to determine what is currently known about the health and psychosocial impacts of hepatitis C along the trajectory from exposure to ongoing chronic infection, and to identify what knowledge gaps remain.

**Methods:**

PubMed, Current Contents and PsychINFO databases were searched for primary studies published in the ten years from 2000–2009 inclusive. Two searches were conducted for studies on hepatitis C in adult persons focusing on: outcomes over time (primarily cohort and other prospective designs); and the personal and psychosocial impacts of chronic infection. All retrieved studies were assessed for eligibility according to specific inclusion/exclusion criteria, data completeness and methodological coherence. Outcomes reported in 264 included studies were summarized, tabulated and synthesized.

**Results:**

Injecting drug use (IDU) was a major risk for transmission with seroconversion occurring relatively early in injecting careers. Persistent hepatitis C viraemia, increasing age and excessive alcohol consumption independently predicted disease progression. While interferon based therapies reduced quality of life during treatment, improvements on baseline quality of life was achieved post treatment – particularly when sustained viral response was achieved. Much of the negative social impact of chronic infection was due to the association of infection with IDU and inflated assessments of transmission risks. Perceived discrimination was commonly reported in health care settings, potentially impeding health care access. Perceptions of stigma and experiences of discrimination also had direct negative impacts on wellbeing and social functioning.

**Conclusions:**

Hepatitis C and its management continue to have profound and ongoing impacts on health and social well being. Biomedical studies provided prospective information on clinical aspects of infection, while the broader social and psychological studies presented comprehensive information on seminal experiences (such as diagnosis and disclosure). Increasing the focus on combined methodological approaches could enhance understanding about the health and social impacts of hepatitis C along the life course.

## Background

Hepatitis C infection is now acknowledged as an issue of major public health importance for most countries in the world [1]. In Australia, hepatitis C is one of the most commonly notified communicable diseases with an estimated prevalence approaching 1.5% [2]. Nationally, there have been over 230,000 notifications since 1995 (when mandatory notification was established in all jurisdictions) and the annual number of new notifications has averaged around 12,000 for the past five years [3]. Recent estimates put the global hepatitis C prevalence at around 2.4%, with up to 170 million people now thought to be chronically infected [4]. Between 70% and 85% of those initially infected fail to clear the virus [5-7]. Hepatitis C treatments are available, but uptake remains low even in countries such as Australia, where treatment is available at low cost to eligible patients [8,9]. Thus, most people with hepatitis C infection face the prospect of lifelong chronic infection.

The personal impacts of a diagnosis of hepatitis C infection are known to be significant. The direct effects of the virus and its management on wellbeing can lead to people making significant lifestyle changes including reducing work hours or alcohol consumption [10,11], which may in turn influence economic status and social participation. There can be negative social implications for people living within the context of a broader community who may be largely uneducated about hepatitis C transmission. In most cultures around the world there is prevailing marginalization of people who inject drugs – the major risk factor for infection [12-14]. Disclosure of hepatitis C status can result in alienation from family and friends as well as perceived and actual discrimination in health services and workplaces [12]. As with other chronic diseases, experiences of diagnosis and management are shaped by a multitude of physical and psychosocial forces. Such forces influence the dynamics of adaptation to illness and impact on well being, often without direct linearity, over time [15]. Given the size of the affected population, having an understanding of these forces in relation to adapting to, and living with, hepatitis C is critical for effective health policy planning.

Many countries have developed, or are currently developing, national strategies aimed at reducing the harms associated with hepatitis C. For example, the soon to be released *National Liver Strategy* represents the second strategy for the United Kingdom, while the *Third National Hepatitis C Strategy* (covering the period 2010 to 2013) has now been implemented in Australia. Making these strategies relevant to the vast majority of those affected by hepatitis C, particularly those for whom treatment does not appear to be either useful or desirable, would seem to require a greater understanding of the ongoing impacts of hepatitis C diagnoses. Using a systematic approach, we undertook a scoping exercise of the biomedical and social literature published in the ten years from 2000 on the ongoing health and social consequences of diagnoses with hepatitis C infection. We searched for studies on hepatitis C in adult persons in which the health or social outcomes of infection were investigated (including studies of hepatitis C transmission). We specifically searched for cohort studies (and other longitudinal designs) as well as studies using qualitative methodologies. The objective of this study was to determine what is currently known about the health and social impacts of hepatitis C along the trajectory from exposure to ongoing chronic infection, and to identify what knowledge gaps remain.

## Methods

We developed an inclusive search strategy aimed at including studies using both qualitative and quantitative approaches. We searched the health literature in the PubMed (incorporating Medline), Current Contents and PsychINFO data bases for the years 2000 to 2009 inclusive, using two different strategies. The first search used the terms “(hepatitis C OR HCV) AND (cohort study OR follow up study OR longitudinal study OR prospective study OR retrospective study OR concurrent study)”; and the second search used the terms “(hepatitis C OR HCV) AND (quality of life OR social impact OR socioeconomic impact OR psychological well being).” We limited the search to primary studies in adults and excluded investigations that focused solely on evaluating the performance of diagnostic tools and experimental interventions without providing retrievable data on patient health outcome beyond biochemical or serological endpoints (Table 1).

**Table 1:** Example of electronic search strategies used – limits: Humans, Male, Female, English, All Adult: 19+ years, Publication Date from 2000/01/01 to 2009/12/31

	**Search strategy 1**	**Search strategy 2**
Search terms:	“(hepatitis C OR HCV) AND (cohort study OR follow up study OR longitudinal study OR prospective study OR retrospective study OR concurrent study)”	“(hepatitis C OR HCV) AND (quality of life OR social impact OR socioeconomic impact OR psychological well being).”
Number of studies identified in total:	1608	335
Number of studies excluding reviews:	1592	320
Number of studies excluding experimental trials	570	299

To enhance the relevance of reviewed studies to the current context of hepatitis C and its management, we excluded studies published before January 2000, limited to the end of the last full calendar year at the time of the review – December 2009. Resource and time restrictions limited the search to English language journal publications. Studies were excluded if specific data on hepatitis C outcomes were not retrievable (for example, in a study of liver disease in general). We also excluded studies that solely focused on quantifying hepatitis C treatment side effects, unless the personal impact of the symptoms was also investigated – for instance, their effect on quality of life, social function or reported health status. The specific inclusion and exclusion criteria were as follows:

Inclusion criteria

#8226;Primary studies

#8226;Adult participants

#8226;Hepatitis C specific data provided

#8226;Publication between January 1, 2000 and December 31, 2009

#8226;English language

Exclusion criteria

#8226;Data not provided on participant health outcome (beyond serologic endpoints)

#8226;Relevant outcome data not retrievable

#8226;Non-English language

#8226;Previously published analyses of the same data

All identified abstracts were scanned, before relevant articles were retrieved and reviewed. When the same cohort was described in successive publications, only the latest publication was reviewed except where subsequent analyses focused on different outcomes. The reference lists of reviewed articles were also scrutinized for any additional items relevant to the review. Records of all articles retrieved were stored and managed in EndNote (version X3.0.1).

As this review aimed to determine if it was possible to build a picture of the trajectory of a hepatitis C diagnosis by summarizing the evidence from a broad range of eligible studies (using both quantitative and qualitative approaches), the quality assessment was restricted to the completeness of information provided by the authors. Studies were required to provide sufficient data to characterize the participants (e.g. by age, sex, and population group), the follow up period (if relevant to the design), the methodological approach (including any instruments utilized), and outcomes (including appropriate measures of effect such as Relative Risk and Hazard Ratios, in the case of quantitative designs). Inclusion and exclusion criteria were developed by consensus of the authors as were the categories assigned to the reviewed studies. Eligible studies were then summarized and reviewed by the research team, before coding into the specified categories for narrative synthesis. See Additional file 1 for the full list of included articles, as well as their tabulated summaries.

## Results

**Figure 1:**
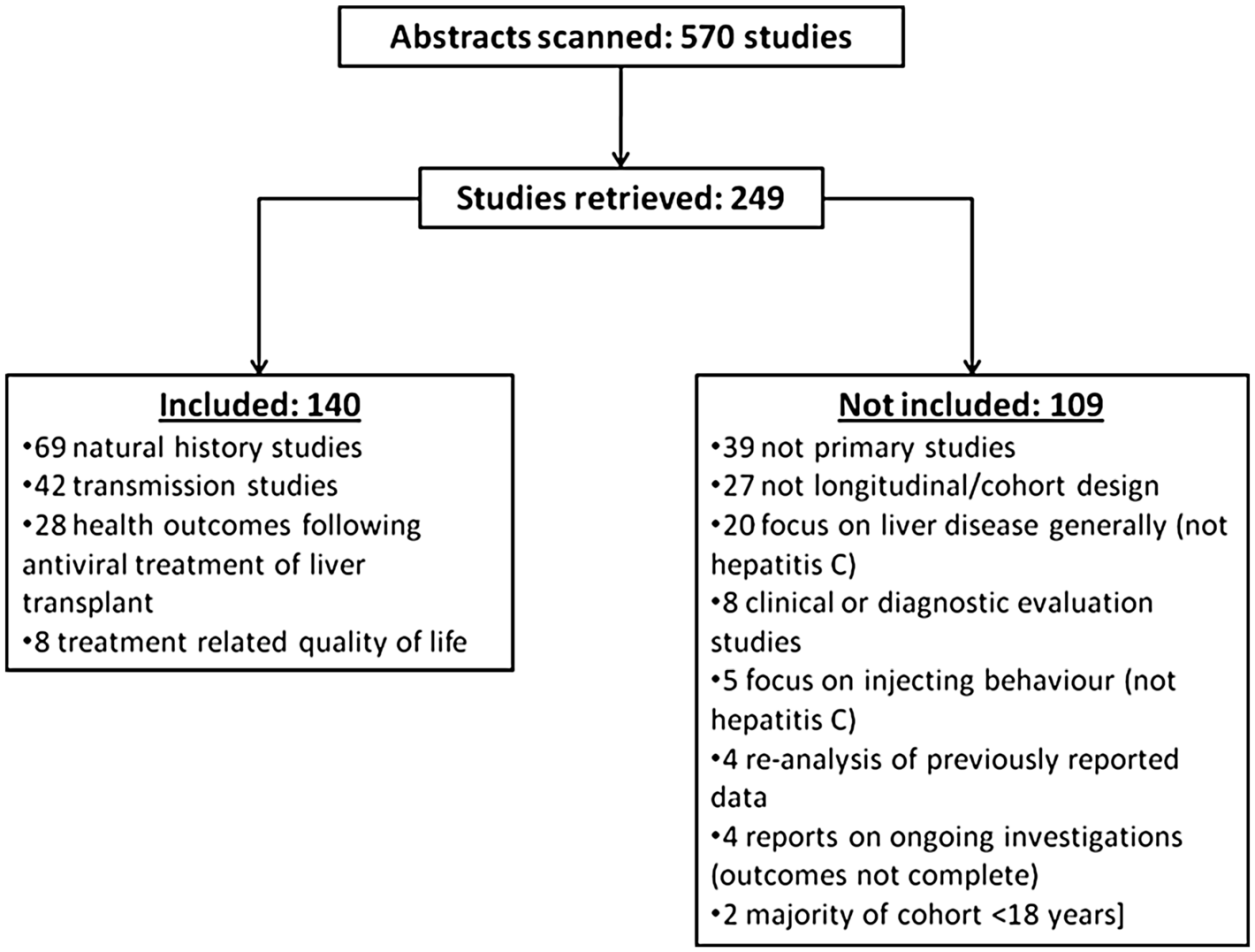
Flow chart of included and excluded studies identified in search strategy 1.

We identified 140 cohort (or follow up) studies with our first strategy (see Figure 1) and 133 studies with our second strategy. With the exception of eight studies (all of which investigated health related quality of life), there was little duplication between the results obtained using the two sets of search terms.

### Temporal experience of hepatitis C

The cohort studies provided comprehensive information on the clinical and epidemiological impacts of hepatitis C over time and covered the broad categories of: transmission; natural history; health related quality of life during the course of antiviral treatment; and health outcomes after antiviral treatment or liver transplant (see Table 2). With the exception of eight studies in people undergoing antiviral treatment, no cohort studies investigated the personal or social experiences, of living with hepatitis C.

**Table 2:** Identified hepatitis C follow-up studies published in 2000 to 2009 – n = 140

*Category *(number of studies)*	**Country of study *(number of studies)***	**Populations or study focus *(number of studies)***	**Follow up period range**	**Sample size range**
Transmission (*42*)	Australia (*5*); Brazil (*1*); Canada (*5*); Chile (*1*); China (*2*); Denmark (*1*); Egypt (*3*); Germany (*1*); Italy (*3*); Japan (*3*); Netherlands (*1*); Scotland (*1*); Spain (*1*); Switzerland (*1*); Thailand (*1*); UK (*3*); US (*9*)	Endoscopy patients (*1*); general populations (*5*); hemodialysis patients (*5*); injecting drug users (*18*); men who have sex with men (*2*); people with HIV (*3*); perinatal populations (*4*); prisoners (*4*)	0.3 to 11 years	18 to 6,412
Natural history (*69*)	Australia (*2*); Canada (*1*); China (*1*); Denmark (*2*); Europe – 8 centres (*2*); France (*1*); Greece (*1*); Ireland (*1*); Italy (*9*); Japan (*19*); Netherlands (*2*); Spain (*6*); Sweden (*3*); Switzerland (*1*); Thailand (*1*); Turkey (*1*); Taiwan (*2*); UK (*2*); US (*12*)	Hepatitis C mono-infection, with/out co morbidity (*41*); hepatitis B and C co-infection, or cf. Mono-infection outcomes (*15*); hepatitis C and HIV co-infection, or cf. Mono-infection outcomes (*11*); hepatitis C, B and HIV co-infection, or cf. mono –infection outcomes (*2*);	1.0 to 35 years	17 to 474,369
Antiviral treatment related quality of life (*8*)	Italy (*1*); Sweden (*1*); Turkey (*1*); US (*5*)	Patients undergoing treatment (*4*); patients assessed pre- and post-treatment (*4*)	Up to 1.4 years	18 to 1,144
Health outcomes post treatment or other management (*28*)	Canada (*4*); Europe – 8 centres (*1*); France (*1*); Greece (*1*); Italy (*2*); Japan (*7*); Norway (*1*); Switzerland (*2*); Thailand (*1*); US (*8*)	Hepatitis C mono-infection and antiviral treatment (*15*); hepatitis C, hepatitis B and/or HIV co-infection – hepatitis C antiviral treatment response (*4*); post liver transplant patients, with/out hepatitis C antiviral treatment (*9*)	0.5 to14.4 years	45 to 13,855

*seven studies are included in more than one category.

#### Transmission of Hepatitis C

We identified 42 cohort studies investigating the transmission of hepatitis C in various community and hospital based populations. Eighteen of these followed up community-based injecting drug users (IDU) and two studies also included non-injecting participants (see Additional file 1: Table S1). In thirteen, the IDU cohort was seronegative for hepatitis C at baseline, with rates of new infection ranging from 8.1 to 45.8 per 100 person-years. While all reported incidence rates were significantly higher than in non-IDU, rates appeared to be unrelated to the period of follow up. This may be due to infection of susceptible populations relatively early in their injecting careers. Roy et al. [16] noted that around 50% of a cohort of IDU in Canada had seroconvert within the first four years of injecting, and Maher et al. [17] found that being within the first year of injecting independently predicted hepatitis C seroconversion after adjusting for a range of demographic factors and risk behaviours. Re-infection rates among IDU previously achieving viral clearance tended to be lower than new infection rates – suggesting a role for host related factors.

Twenty four studies investigated incidence rates in the general population and its subpopulations (see Additional file 1: Table S2). Studies undertaken in general population samples in Chile, Italy and Japan indicate tha the incidence of hepatitis C general populations overall was low compared to IDU populations – ranging from 0.01 to 0.4 per 100 person years in four identified studies. A higher general population rate was reported by Grebely et al. [18] – 7.4 per 100 person years – who sampled an inner city population that included a high proportion of IDU. Reported rates were higher in renal patients undergoing long term hemodialysis in some countries, although reductions were observed in Italy and Germany in line with improving infection control practices.

There is evidence that perinatal transmission is associated with maternal hepatitis C viraemia, and is significantly more frequent in maternal HIV co-infection. While up to 31 percent of babies born to mothers with hepatitis C were reported to be seropositive shortly after birth, Sbebl et al. [19] found that only 2.4% remain so by three years of age. Hepatitis C transmission in people already infected with HIV, and in HIV negative men who have sex with men, was relatively low – and occurred mainly through injecting drug use. Four studies in the UK and Australia demonstrated an elevated transmission rate in prisoner populations – particularly in prisoners reporting injection drug use whilst incarcerated. Dolan et al. [20] estimated a rate of 21.3 per 100 person years in imprisoned heroin users, while a rate of 4.6 per 100 person years was observed in the South Australian prison population [21] – although community exposure couldn’t be ruled out in the latter study.

#### Natural history of chronic hepatitis C infection

We identified 69 cohort studies investigating the natural history of chronic hepatitis C infection (see Additional file 1: Table S3). Of these, 42 studies focused on health outcomes for people mono-infected with hepatitis C, while the remainder (27 studies) compared outcomes in hepatitis C, hepatitis B or HIV mono- and co-infection. Mortality, disease progression and development of hepatocellular carcinoma (HCC) were outcomes investigated by the majority of all studies, and a small number focused on viral clearance. Two studies investigated behavioral outcomes – one looking at clinical factors associated with treatment uptake and the other looking at general practitioner (GP) patterns of management and referral to specialists in hepatitis C.

The studies in people with untreated chronic hepatitis C monoinfection had follow up periods ranging from one to 35 years in samples of clinical groups as small as 17 to sample sizes approaching half a million from large clinical data bases. The evidence for fibrotic changes and development of liver cirrhosis, development of HCC and increased liver-specific mortality in chronic hepatitis C over time was relatively consistent. Several studies found that the more serious complications of chronic infection were predicted by the presence of persistent viraemia, moderate to high alcohol consumption and increasing age. Age was not as important in a study in elderly Italians with chronic hepatitis C, which found deaths to non-liver related causes occurred more frequently than liver-specific deaths over ten years of follow up. Studies investigating health outcomes for people with persistently normal alanine amino transferase (ALT) levels suggest that liver cirrhosis and HCC do occur, but the frequency and rate of disease progression tends to be low relative to those with consistently high ALT levels. Despite the apparent importance of this indicator, Yawn et al. [22] found that less than half of primary care physicians monitored ALT in their patients with hepatitis C. While unlikely to have direct impact on the experience of living with hepatitis C, a lack of ongoing ALT monitoring might eventually have implications for health outcome in their patients. Yawn et al. also found there was only limited and inconsistent management and referral with respect to potential accelerators of progression such as excessive alcohol consumption and viral co-infections.

Twenty seven studies compared the longer term effects of chronic mono-infection with hepatitis C and hepatitis B or HIV, and/or the impact of co-infection with any of these. Fifteen studies investigated the independent or combined effects of hepatitis B and C for between 3.4 and 23 years of follow up. While studies varied in the order of risk for hepatitis B or C monoinfection, co-infection was relatively consistently associated with greater incidence of HCC and lower survival than mono-infection. In their 23 year follow up study, Zampino et al. [23] found that earlier age at infection was associated with a lower disease progression in both mono- and co-infected patients.

Eleven studies were in cohorts infected with either HIV or hepatitis C or both. Most of these compared the independent effects of infection with each virus – with outcomes such as AIDS, liver cirrhosis, and overall and specific mortality. An exception was Grebely et al. [24] who investigated hepatitis C viral clearance (rather than disease outcome) in a community sample in Canada, and found that co-infection with HIV and continuing injecting drug use were associated with hepatitis C viral persistence. In other studies, HIV-hepatitis C co-infection was found to have a synergistic effect in which the progression to AIDS and end stage liver disease and liver-related death (mostly due to HCC) was accelerated. Improvements in survival were calculated for HIV patients in hepatitis C-related end stage liver disease under active anti-retroviral treatment (HAART), although this finding was not supported by other studies in this group (see García-Garcia et al. [25] for example). In their study of antiviral treatment initiation for hepatitis C in Denmark, Hansen et al. [26] found that 33% of patients commenced treatment within five years and this was predicted by higher ALT, HCV genotype 2 or 3 (the most favourable for successful treatment outcome) and HIV negative status.

We identified two cohort studies investigating the combined and/or independent effects of infection with hepatitis B, hepatitis C and HIV. Bonacini et al. [27] found liver-related mortality in HIV patients was increased regardless of the hepatitide with which they were co-infected, and was more common when both were present. In a cohort of haemophilia patients with HIV infection, Melendez-Morales et al. [28] found an 11-fold increase in hepatitis C viral clearance in the presence of hepatitis B. The authors suggest that this may be due to ‘mutual interference in viral replications’ – as is thought to occur in the presence of hepatitis D.

#### Health related quality of life during the course of antiviral treatment

Eight cohort studies investigated the impact of hepatitis C treatment on health related quality of life (QoL) (see Additional file 1: Table S4). Six of these studies used the 36-item Medical Outcomes Study Short-Form 36 (SF-36) or the 12 item version (SF-12), in wide use around the world to measure health status across eight domains relevant to physical, mental and emotional health. The SF-36 and various measures to establish depression levels and other social and emotional dimensions, uniformly demonstrated reduced health related quality of life during treatment but improving post treatment – particularly when sustained viral responses were achieved. Evon et al. [29] found the prevalence of depression was relatively high in patients prior to treatment (12%) and this predicted early exit from treatment. Depression newly diagnosed *during* treatment had a lesser impact on early exit.

#### Health outcomes after antiviral treatment or liver transplant

Twenty eight studies investigated health outcomes following interferon based treatments (generally in combination with Ribavirin) or after liver transplant (see Additional file 1: Table S5). Fifteen studies were in patients with no viral co-infection who were followed up from six months to 14 years after treatment. In most populations, sustained viral responses were attained in between 20% and up to 80% depending on the viral genotype (with genotypes other than 1 and 4 considered the most favourable), and sustained viral responses were associated with a lower incidence of complications such as HCC and lower mortality. Dalgard et al. [30] demonstrated that the comparable outcomes were achievable in people who inject drugs, despite ongoing concerns about compliance with treatment schedules and ongoing drug use.

Four studies investigated treatment-related outcomes in hepatitis C patients co-infected with either hepatitis B or HIV. Ikeda et al. [31] found that any viral response to hepatitis C treatment (transient or sustained) was protective for the development of HCC, but not in the case of co-infection with hepatitis B. Co-infection with HIV appeared to be a barrier to evaluation for, and uptake of, hepatitis C treatment although sustained response rates (per genotype) have been reported to be similar to those seen in hepatitis C mono-infection. Furthermore, hepatitis C disease progression may be slowed with treatment even when sustained response is not achieved.

We identified nine cohort studies investigating health outcomes after liver transplantation. Four of these studies compared health outcomes in organ recipients with and without markers for hepatitis C. In all cases, hepatitis C was associated with lower survival and reduced overall health and function. In their seven-year retrospective study of patients following hepatitis C related liver transplants, Gallegos-Orozco et al. [32] estimated a median survival time of 3.5 years. According to Bizollon et al., [33] sustained response to hepatitis C treatments provided post-transplantation is associated with improved graft survival although not necessarily with improved survival.

### The personal and psychosocial impacts of hepatitis C

**Figure 2:**
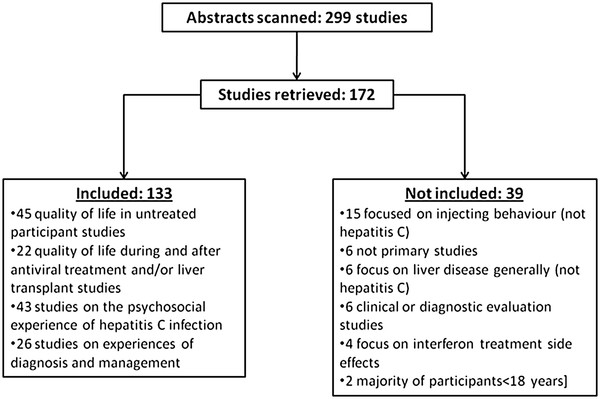
Flow chart of included and excluded studies identified in search strategy 2.

Our second strategy identified 133 studies investigating the personal experience and social impacts of hepatitis C – in terms of individual wellbeing and psychosocial function (see Figure 2). The studies could be grouped into four broad categories: two categories focusing on either the QoL impacts of ongoing infection or its treatment; one category exploring the psychosocial experience of living with hepatitis C; and a final category focusing on experiences of diagnosis and management of hepatitis C (see Table 3). Most of the QoL studies used a quantitative approach, while qualitative approaches were more commonly taken in the broader social research. Very few studies were longitudinal in design, with the majority focusing on analyzing data collected at a single point in time.

**Table 3:** Identified hepatitis C social research studies published in 2000 to 2009 – n = 133

*Category *(number of studies)*	**Country of study *(number of studies)***	**Populations or study focus *(number of studies)***	**Approach used *(number of studies)***
Quality of life in people with untreated hepatitis C (*45*)	Australia (*6*); Austria *(2)* Brazil (*2*); Canada (*4*); Egypt (*1*); France (*1*); Germany (*3*); Ireland (*2*); Italy (*2*); Mexico (*1*); Netherlands (*1*); Norway (*2*); Serbia (*1*); Spain (*1*); Sweden (*1*); Switzerland (*1*); Taiwan (*1*); Turkey (*3*); US (*10*)	Community and clinic populations (*26*); injecting drug users (*2*); hepatitis B and/or HIV co-infection (*7*); people with various stages of disease progression (*4*); people with comorbid conditions (*3*)	Quantitative approaches using surveys incorporating physical and mental health status instruments such as the SF-36 or 12, Beck Depression Inventory (*45*)
Quality of life in people during or after antiviral treatment/transplant for hepatitis C (*22*)	Australia (*1*); Canada (*1*); Germany (*1*); Italy (*3*); Sweden (*1*); Taiwan (*3*); Turkey (*2*); UK(*1*); US (*9*)	During antiviral treatment (*13*); after antiviral treatment (*5*); after liver transplantation (*3*)	Quantitative approaches using surveys incorporating physical and mental health status instruments such as the SF-36 or 12, Beck Depression Inventory (*20*)
			Qualitative approaches involving semi-structured or in-depth interviews (*2*)
Psychosocial experience of living with hepatitis C (*43*)	Australia (*6*); Canada (*5*); France (2*1*); Germany (*2*); Ireland (*1*); Italy (*1*); Scotland (*1*); Turkey (*1*); UK (*2*); US (*6*)	Social functioning (*6*); life style (*6*); general health and well-being (*9*);	Quantitative approaches using surveys incorporating clinical and psychosocial data (*26*)
			Qualitative approaches involving semi-structured and/or in-depth interviews, focus groups and ethnographic observation (*14*) Mixed methods using semi-structured interviews and survey techniques (*3*)
Experience of diagnosis and management of hepatitis C (*26*)	Australia (*9*); Australia & New Zealand (*1*); Canada (*3*); France (*1*); Ireland (*5*); Japan (*1*); New Zealand (*1*); Pakistan (*1*); Turkey (*1*); UK (*3*); US (*15*)	Diagnosis impact (*6*); stigma & discrimination (*5*); health service access (*10*); treatment decisions (*5*)	Quantitative approaches using surveys incorporating clinical and psychosocial data (*11*)
			Qualitative approaches involving semi-structured and/or in-depth interviews, focus groups or daily participant recordings (*13*)
			Mixed methods using semi-structured interviews and survey techniques (*2*)

*three studies are included in more than one category.

#### Quality of life in untreated hepatitis C

Sixty six studies investigated quality of life (QoL), including eight cohort studies in people undergoing treatment also identified in the biomedical literature. Forty five of these studies measured QoL impacts in untreated chronic infection in community-based and clinic populations, former or current IDU, people with viral co-infections, patients with various stages of disease progression, and patients with co morbidities (see Additional file 1: Table S6). Again, the SF-36 (or SF-12) was the main instrument used to assess health related QoL, and several studies used other validated instruments measuring mental health and psychosocial status. The studies provide relatively consistent evidence of reduced QoL in untreated hepatitis C in most populations. The exception was provided by Bailey et al., [34] who observed moderate to high levels of QoL in US hepatitis patients, upon whom the main negative impact was uncertainty surrounding prognosis. In Australia, Thein et al. [35] found little difference in QoL between HIV and hepatic C mono-infected, co-infected and un-infected patients – although the authors pointed out the numbers in each group may have been too small to demonstrate clear differences.

There was substantial variation among studies about the relative impact of a range of co-factors on QoL. Several studies concluded reduced QoL in hepatitis C was independent of disease activity, liver histology and drug and alcohol use. Gunasekera et al., [36] however, found that QoL was reduced in hepatitis C patients recruited from rural drug and alcohol services when compared to rural primary care or hepatitis C centres. Continued drug use was associated with reduced QoL in two other studies, but not in others analyzing the role of substance use. Two studies led by Kramer [37,38] both found reduced QoL in hepatitis C which was associated with fatigue, but not with observed slight neuro-cognitive impairment. Fatigue and depression accounted for the majority of reduced QoL in a study of HIV-hepatitis C coinfected patients in France [39].

Several studies proposed an emotional or psychosocial basis for reduced QoL. Dalgard et al. [40] and Shwarzinger et al. [41], for example, found that awareness of hepatitis status explained much of the reduction in QoL. Depression and psychiatric co morbidities were found to be important in some studies, although others found that the co-existence of mental illness failed to fully explain the QoL differentials in those with and without hepatitis C. While several studies found little association between QoL and ALT or liver fibrosis in clinic populations, *advanced* liver disease did appear to be closely correlated with reduced QoL in hepatitis C.

#### Quality of life related to hepatitis C treatment

Twenty two studies investigated health related QoL in patients undergoing antiviral treatment or liver transplantation, with the majority using the SF-36 (see Additional file 1: Table S7). The studies were mainly cross-sectional, with seven utilizing a follow up design (as previously described). Most of the eleven studies in patients undergoing interferon-based antiviral treatment noted further reductions in health related QoL during the treatment, with depression playing a significant role. Improvements were noted post treatment in most studies, particularly where sustained virological clearance was achieved. Fontana et al. [42] compared QoL in patients who were accessing treatment and those who were not. They found that emotional distress, which was strongly associated with reduced QoL, was highest in the untreated group – particularly in those who were anticipating a fatal outcome to their chronic hepatitis C infection. One study found no difference in QoL between treated and untreated groups but did find that concurrent treatment had a negative impact on cognitive abilities [43]. Studies of patients post treatment suggest that the benefits of successful treatment can be sustained over time, as can the deleterious effects of less successful treatment. In their study of untreated patients, patients who had relapsed after treatment and patients who did not respond to treatment, Taliani et al. [44] found that non-responders had the lowest QoL. Low quality of life scores were independently associated with co morbidity and non-response to treatment. They suggest that treatment expectations may be important modifiers of QoL.

Three studies investigated QoL following hepatitis C related liver transplantation. Two quantitative studies found significant impairments in QoL in recurrent hepatitis C (occurring in 36% to 62% of the study populations) in the years following transplants, while non-recurrence was associated with improved functional performance and quality of life over time. In a qualitative study involving in-depth interviews with eight post transplant patients, Dudley et al. [45] concluded that stigma and disease uncertainty in hepatitis C continues after liver transplants and may further impact adversely on QoL in hepatitis C patients.

#### Psychosocial experience of living with hepatitis C infection

We identified 43 studies investigating the impact of hepatitis C on social functioning, lifestyle, health and well-being (see Additional file 1: Table S8). In a majority of studies, diagnosis with hepatitis C was reported to have profound impacts on social functioning. The perceived stigma associated with infection led to high levels of anxiety, and exaggerated fears of transmission and was the main driver of social isolation and reduced intimacy in relationships [46-48]. Grundy and Beeching [46] found that fear of transmission – particularly in intimate relationships, child birth and child rearing – led to concerns about being able to fulfil gender roles in a group of women with hepatitis C. There is further evidence that the social experience of living with hepatitis C differs between women and men. For instance, Temple-Smith et al. [49] found substantial gender differences in health seeking behaviours and notions of social support.

Fear of transmission, where the perceived risk is often unnecessarily inflated, also appeared to result in unnecessary changes in everyday practices, such as refraining from sharing towels and drinking glasses or not taking part in food preparation [50]. Changes in intimate and/or sexual practices, sometimes exhibiting as sexual dysfunction, have been observed in association with depression and anxiety about transmission [51]. Other lifestyle impacts of hepatitis C include reductions in alcohol consumption, reductions in smoking and modification of dietary intake [52]. In the context of competing priorities including housing, employment and legal implications of injecting, hepatitis C was viewed to be of less consequence by some study participants who injected drugs, but modification of risk behaviours (such as cleaning of syringes and spoons, and less sharing of injecting paraphernalia) was reported [53,54].

Infection with hepatitis C has been found by a number of studies to have substantial impacts on health and well-being, often in relation to fear and uncertainty about prognosis [55]. One study found that feelings of hopelessness in conjunction with uncertainty were experienced by people with either hepatitis C or HIV, but tended to be heightened in hepatitis C [56]. Perceived stigma and discrimination impeded adaptation to the hepatitis diagnosis and was a common source of anxiety in people with chronic hepatitis C [56,57]. Hopwood and Treloar [58] found that the psychosocial stress associated with hepatitis C was less in those with a history of IDU, which may arise from greater resilience in coping with hepatitis C stemming from experiences of marginalization for people who inject drugs. A number of studies investigated specific symptoms of hepatitis C infection – with fatigue being the most common, followed by depression and other mental health issues. Bodily pain, particularly in the form of myalgia and a degree of cognitive impairment was also identified by several authors. The majority of studies found symptoms were independent of disease activity or disease severity, but associated with depression, anxiety and other psychosocial factors. The direction of causation might prove difficult to untangle, particularly where specific symptoms are tightly clustered. Golden et al., [59] for instance found that mood disorders were highly prevalent in hepatitis C, and depression was associated with experiences of illness – including stigma, poor adjustment to the diagnosis and physical symptoms. There were three studies that suggested a biological cause for a slight cognitive impairment observed in hepatitis C [60-62].

#### Responses to diagnosis and management of hepatitis C

Finally, we identified 26 studies investigating experiences related to diagnosis and treatment (see Additional file 1: Table S9). With some degree of overlap, these studies focused on the immediate impact of diagnosis and perceptions of stigma and discrimination in relation to it, accessing health services and making decisions about treatment. Diagnosis with hepatitis C could be a stressful event, characterized by feelings of shock and devastation that transitioned into enduring emotional, psychosocial and even physical effects [63]. For some, the time of diagnosis was an event equivalent to the stress of events such as moving cities, losing a job, marital breakdown and divorce [64]. Some studies, however, described a more dynamic response that was mediated by changes in social context [65]. For instance, the threat of HIV and issues related to substance use was considered a higher priority in some groups [66].

The perception of stigma is generally an internalized phenomenon resulting from individually held understandings and interpretations, or arising in response to actual or perceived discrimination. For instance, study participants described perceptions of stigma resulting from feelings of contamination and fear of disclosure to others, from whom they anticipated rejection, much of which was on the basis of fear of disease transmission [67]. Golden et al. [68] found perceived stigma was associated with decreased acceptance of illness, decreased social adjustment and increased reported symptoms in hepatitis C.

Discrimination can flow from the beliefs and attitudes of others and ultimately shape perceptions of stigma through the differential treatment of people with particular conditions. As Paterson et al. [69] describe, how illnesses are constructed by health providers influences not only the care offered, but also feeds into the self perception of the affected person. Many study participants reporting negative experiences in health care settings in relation to perceived discrimination. In one study of over 500 people with hepatitis C, 65% reported having experienced health care discrimination, which was associated with pessimism about future health and decreased social interaction among other things [70]. Perceived discrimination was also found to be a significant barrier to health treatment access, to the extent that refusal of treatment by providers was reported [71]. Harris [72] found that reports of refusal or withdrawal of health care were common and contributed to a reluctance to disclose to health professionals even in the context of a perceived obligation to do so.

Five studies investigated treatment decision making, confirming that concern about side effects remains an important reason for declining or adhering to treatment. McNally et al. [8] proposed that confidence in treatment efficacy was the main consideration in deciding to undertake treatment. Underscoring the contextual influences involved in making treatment decisions, Ogawa and Bova [73] suggested that treatment decisions in former IDU were complicated by fears that self injecting interferon could reintroduce the use of syringes in a way that might threaten their control over their injecting drug use. Treloar and Hopwood [74] encountered what they defined as ‘unrealistic optimism’ in both patients and health care providers, in which the applicability of information about possible side effects was underestimated. This may have implications for delay in treatment for mental health issues that might arise during antiviral treatment (Table 4).

**Table 4:** Summary of findings from the biomedical and social literature on the ongoing clinical and psychosocial impacts of diagnoses with hepatitis C infection

**Category**	**Findings**
Transmission	· Strongly associated with injection drug use – likely to occur early in injecting career.
	· Maternal transmission associated with hepatitis C viraemia – more frequent in maternal HIV co-infection.
	· Prisoner populations at enhanced risk for infection.
Natural history	· Chronic infection can progress to fibrotic changes and development of liver cirrhosis, development of hepatocellular carcinoma (HCC) and increased liver-specific mortality
	· Complications of chronic infection predicted by persistent viraemia, moderate to high alcohol consumption and increasing age.
	· Liver cirrhosis and HCC occur in persistently low serum alanine amino transaminase (ALT), but frequency and rate of disease progression low relative to consistently high serum ALT.
	· Hepatitis B co-infection associated with greater incidence of HCC and lower survival than mono-infection with either virus.
	· HIV-HCV co-infection found to accelerate progression to AIDS, end stage liver disease and liver-related death (mostly due to HCC).
Health related quality of life (QoL)	· Relatively consistent evidence of reduced QoL in untreated hepatitis C in most populations.
	· Variation among studies about the relative impact of a range of co-factors on QoL, although disease activity found to be independent of QoL.
	· Interferon-based treatments associated with further reductions in health related QoL, with depression playing a significant role.
	· QoL improves post treatment – particularly if sustained viral responses achieved.
	· QoL benefits of successful treatment can be sustained over time (possibly the deleterious effects).
Health outcomes after antiviral treatment or liver transplant	· In most populations, including IDUs, sustained viral responses attained from 20% to 80% depending on viral genotype (types other than 1 and 4 considered the most favourable).
	· Sustained viral response associated with lower incidence of complications (e.g. HCC and death).
	· Hepatitis C associated with lower survival and reduced overall health and function in organ transplant patients.
Psychosocial experience of living with hepatitis C infection	· Diagnosis with hepatitis C reported to have profound impacts on social function.
	· Perceived stigma led to high levels of anxiety and over-inflated assessments of transmission risks.
	· Fatigue the most common symptom reported, followed by depression and other mental health issues, and myalgia.
	· Symptoms independent of disease activity or disease severity, but reported to be associated with depression, anxiety and other psychosocial factors with some biological mechanisms proposed.
Responses to diagnosis and management of hepatitis C	· Diagnosis with hepatitis C often reported to be a stressful event, potentially mediated by personal and social context.
	· Perceived discrimination reported in multiple settings, including in interactions with health care services.
	· Potentially a barrier to health service access and treatment
	· Fear of side effects reported as a major influence on treatment decisions.

## Discussion

In this paper we reviewed the biomedical and social literature on the ongoing clinical and psychosocial impacts of diagnoses with hepatitis C infection. The published literature provides useful information on selected aspects of living with hepatitis C. Identified cohort studies provided prospective information on clinical aspects of chronic infection in the longer term, with some studies involving very large numbers of patients who were followed up for considerable lengths of time. The social research presented in depth information on some of the social and personal ramifications of living with hepatitis C in the form of comprehensive and contextual ‘snapshots’ of seminal experiences, such as diagnosis and disclosure to others. Yet questions still remain if we are to develop a comprehensive understanding about what happens to people once they are diagnosed with hepatitis C.

The findings summarized here contribute to the knowledge base and could inform the continuing development, and revision, of national strategies aimed at reducing the harms associated with hepatitis C around the world. That the findings are synthesized from a wide range of methodological and discipline related perspectives could potentially enhance their relevance to strategy development and health service planning into the future. Yet the picture still remains fragmented and incomplete. The sometimes substantial impact of hepatitis C (and its management) on QoL has been frequently investigated, but only relatively short term follow up of changes in QoL during the course of treatment has been reported. Over the longer term people may experience fluctuations in disease activity, modification of alcohol or other drug use or changing social circumstances and it is likely that these dynamic forces would influence QoL in a manner that cannot be explored using traditional cross-sectional approaches.

Planning for effective health and community service provision for the growing number of people affected by hepatitis C requires a good understanding of potential trajectories, and the physiological and psychosocial forces underlying them, from hepatitis C diagnosis into the future. As people adapt to their diagnoses (as described in the social research), what proportion go on to find hepatitis has little or no impact on their lives, and what proportion find hepatitis C exerts a significant impact? At which points along their trajectory do people engage with hepatitis C treatment or community support services, and do those services meet their expectations and needs? Do people reporting discrimination at work or in the health system continue to have negative experiences? Is it possible to map improvements in the level of discrimination reported with increasing awareness of hepatitis C in the broader population? How does health status change over time, and how is health status linked with changes in lifestyle factors (drinking, smoking, other drug use) or with the social support people receive, or discrimination they perceive, at different points in time. The research identifies a number of factors that contribute to low uptake of antiviral treatment for hepatitis C. Decisions about treatment may be mediated by a range of health and social factors that may vary over time. It will become important to know how to overcome identified barriers and effectively target information as new treatments [75] and new management strategies become available.

This study used a systematic approach to the search strategy but may have been limited by incomplete retrieval of potentially relevant studies. Resource and time restrictions limited the search to English Language publications and did not allow for a search of unpublished data. It is possible that some ongoing studies of the health and social impacts of hepatitis infection were not identified. While our search of non-intervention studies may have lessened the potential for publication bias, there remains the possibility that our assessment of the combined data may have overestimated the health and social impacts of hepatitis C. To minimize these potential source of bias, we searched three major data bases using an inclusive search strategy, and all potentially eligible studies were retrieved in full.

## Conclusion

This narrative review has provided a useful update on many aspects of living with hepatitis C infection but has also highlighted a number of important research gaps that may have implications for hepatitis C strategy development and implementation around the world. Many of these gaps appear to be due to a methodological and content gulf existing between the biomedical and social research literature. Typically, cohort designs have been used primarily in investigations of clinical or health outcome – with exploration of social parameters primarily focused on identifying their effect on outcome. In broader social research, complex psychosocial phenomena are more comprehensively explored, but temporal change is often not a critical factor. Bridging the research gaps will ultimately require a combined approach, both content and methodology, to study the health and social impacts of hepatitis C along the life course.

## Competing interests

The authors declare that they have no competing interests.

## Authors’ contributions

EM designed the search strategy; retrieved, reviewed, summarized and tabulated the papers; and drafted the manuscript. SM participated in the coordination of the search strategy; reviewed summary data; and critically revised subsequent drafts of the manuscript. JW and MS both participated in the critical revision of the manuscript. All authors read and approved the final manuscript.

## Pre-publication history

The pre-publication history for this paper can be accessed here:

http://www.biomedcentral.com/1471-2458/12/672/prepub

## Supplementary Material

Additional file 1 **Table S1.** Hepatitis C transmission in injecting drug users. **Table S2.** Hepatitis C transmission in various initially hepatitis C negative population groups. **Table S3.** Studies investigating the health outcomes in hepatitis C mono-infection and co-infection with other blood borne viruses. **Table S4.** Health related quality of life associated with antiviral treatment. **Table S5.** Studies investigating the health outcomes after treatment. **Table S6.** Health related quality of life in untreated chronic hepatitis C infection. **Table S7.** Health related quality of life during and after treatment/transplant for hepatitis C infection. **Table S8.** Psychosocial experience of living with hepatitis C infection. **Table S9.** Diagnosis, management and treatment of chronic hepatitis C infection. Click here for file
