# Supplementary material for: The ongoing impacts of hepatitis c - a systematic narrative review of the literature
Source: BMC Public Health. 2012 Aug 18;12:672. doi: 10.1186/1471-2458-12-672 (PMC3505729; doi:10.1186/1471-2458-12-672)
Supplement: Additional file 1 — Table S1. Hepatitis C transmission in injecting drug users. Table S2. Hepatitis C transmission in various initially hepatitis C negative population groups. Table S3. Studies investigating the health outcomes in hepatitis C mono-infection and co-infection with other blood borne viruses. Table S4. Health related quality of life associated with antiviral treatment. Table S5. Studies investigating the health outcomes after treatment. Table S6. Health related quality of life in untreated chronic hepatitis C infection. Table S7. Health related quality of life during and after treatment/transplant for hepatitis C infection. Table S8. Psychosocial experience of living with hepatitis C infection. Table S9. Diagnosis, management and treatment of chronic hepatitis C infection. [file 1471-2458-12-672-S1.pdf]

ADDITIONAL FILE - Included Studies

Table 1: Hepatitis C transmission in injecting drug users

Author (year)	Country	Population (n)	Follow up period	Incidence (per 100 person years)
Backmund et al (2004) [1]	US	(18)	2.8 years (mean)	4.1 (reinfection)
Cox et al (2005)[2]	US	(179)	3 years (median)	27.2
Currie et al (2008)[3]	US	HCV clearers	5.1 years (median)	0.49 reinfection (1.8 in continuing IDU)
Foley & Abou-Saleh (2009)[4]	UK	(95)	1.0 year	9.1
Fuller et al (2004)[5]	US	(114)	0.25 years (mean)	35.9
Grebely et al (2006)[6]	Canada	(1072)	5.2 years (median)	8.1 (re-infections in 1.8)
Hahn et al (2002) [7]	US	(195)	0.25 years (minimum)	25.1
Hallinan et al (2004)[8]	Australia	ORT (195)	2.4 years (mean)	3.8 (1.3 in continuous ORT)
Holtzman et al (2009)[9]	US	(1288)	1.0 year (retrospective)	11.6
Jittiwutikam et al (2006)[10]	Thailand	IDU & non-IDU (515)	2.0 years	44.3 (1.9 in non-IDU)
Judd et al (2005)[11]	UK	(241)	1.0 year	41.8
Kim et al (2009)[12]	Canada	(533)	≥ 0.5 years	16.9
Maher et al (2007)[13]	Australia	(377)	≤ 3.0 years	45.8
Micallef et al (2007)[14]	Australia	(423)	1.0 year (retrospective)	17.0 (re-infections in 31.0)
Page et al (2009)[15]	US	(522)	≥ 0.25 years	26.7 (re-infections in 24.6)
Roy et al (2009)[16]	Canada	(858)	≤ 4.0 years	15.4
Ruan et al (2007)[17]	China	(333)	3.0 years	33.3
van den Berg et al (2009)[18]	Netherlands	non-IDU at entry (352)	6.4 years (median)	0.049 (0.79 in IDU initiates)

Notes : **IDU** – Injecting Drug Use(r); **ORT** – Opium Replacement Therapy

Table 2: Hepatitis C transmission in various initially hepatitis C negative population groups.

Author (year)	Country	Population (n)	Follow up period	Incidence (*per 100 person years)
General population:				
Gonzáles et al (2005)[19]	Chile	(959)	≤ 7.0 years	0.02
**Grebely et al (2009)[20]	Canada	IDU (758)	5 years	7.4
Kondili et al (2002)[21]	Italy	(2032)	7.0 years (median)	0.01
Mazzeo et al (2003)[22]	Italy	(1646)	10.0 years	0.05
Okayama et al (2002)[23]	Japan	(973)	11 years	0.36
Haemodialysis patients:				
**Di Napoli et al (2006)[24]	Italy	(6412)	9.0 years	2.0

Author (year)	Country	Population (n)	Follow up period	Incidence (*per 100 person years)
Furusyo et al (2001)[25]	Japan	(169)	6.6 years (mean)	2.6% per year
Kamagai et al (2005)[26]	Japan	(2114)	3.5 years	0.3
Moreira et al (2003)[27]	Brazil	(240)	1.0 year	3.7
Ross et al (2009)[28]	Germany	(1986)	1.0 year	0.0
Medical procedure:				
Mikhail et al (2007)[29]	Egypt	Endoscopy (149)	0.8 years	2.7
Perinatal populations:				
Mast et al (2005)[30]	US	Live births (244)	5.0 years	5% infants of HCV-PCR$^+$ mothers (0 in PCR$^-$); 25% HIV+ mothers.
Mok et al (2005)[31]	UK	Live births (54)	0.25 years	31% infants
Saleh et al (2008)[32]	Egypt	Mothers (2,171)	2.2 years	0.5 (post partum); 1.2 (peri-natal)
Sbebl et al (2009)[33]	Egypt	HCV$^+$ mothers (329)	3 years	2.4% infants
People with HIV:				
Augenbraun et al (2003)[34]	US	Females (1517)	3.5 years (mean)	0.3
Palacious et al (2009)[35]	Spain	(1231)	8.1 years (mean)	0.2
Rauch et al (2005)[36]	Switzerland	(3327)	4.4 years (median)	0.64 (7.4 in IDU)
Men who have sex with men:				
Alary et al (2005)[37]	Canada	(1,054)	2. 5 years (mean)	0.04 (only in one IDU)
Ruan et al (2009)[38]	China	(507)	1.0 year	0.2
Prisoners:				
Champion et al (2004)[39]	Scotland	(307 males)	0.5 years	3.3
Christensen et al (2000)[40]	Denmark	(158 males)	0.5 years (median)	25.0
Dolan et al (2005)[41]	Australia	(382 male heroin users)	4.2 years (median)	21.3
Miller et al (2009)[42]	Australia	(148)	0.3 years (median)	4.6

*****unless otherwise indicated
****** study also included in other study category

Table 3: Studies investigating the health outcomes in hepatitis C mono-infection and co-infection with other blood borne viruses.

Authors (year)	Country	Population (n)	Follow up period	Outcomes measured
Hepatitis C monoinfection:				
Bruno et al (2007)[43]	Italy	Cirrhosis patients (163)	10.7 years	HCC, mortality.
*Bruno et al (2009)[44]	Italy	Cirrhosis patients (352)	14.4 years	Progression, HCC, mortality.
Casiraghi et al (2004)[45]	Italy	Blood recipients (31)	35 years	Progression, fibrosis.

Authors (year)	Country	Population (n)	Follow up period	Outcomes measured
Delladetsima et al (2006)[46]	Greece	Renal patients (17)	7.2 years	Progression, fibrosis.
*Di Napoli et al (2006)[24]	Italy	Renal patients (6412)	9.0 years	Cumulative survival
Federici et al (2006)[47]	Italy	Blood disorderspatients (696)	6 years	HCV, disease activity, cirrhosis, HCC.
Fong et al (2009)[48]	US	Heart transplant patients (10,630)	≤5 years (retrospective)	Survival.
Forns et al (2001)[49]	Spain	HCV patients (114)	27 years (retrospective)	Cirrhosis, HCC, survival.
Harris et al (2006)[50]	UK	Blood recipients (1,399)	16 years (lookback)	All cause & liver mortality.
Hirofumi et al (2009)[51]	Japan	HCV patients (1,125)	8.2 years	All cause & liver mortality.
Hui et al (2007)[52]	China	HCV patients with normal ALT (82)	8.1 years	Fibrosis, disease activity.
*Ikeda et al (2009)[53]	Japan	Elderly HCV patients (1,917)	6 years	HCC & survival.
Jauncey et al (2004)[54]	Australia	Newly infected IDU (57)	1.8 (retrospective)	Viral clearance.
Keating et al (2005)[55]	Ireland	Newly infected IDU (289)	2 years	Viral clearance.
Kumada et al (2009)[56]	Japan	HCV patients with normal ALT (398)	8.8 years	HCC.
Martinot-Peignoux et al (2001)[57]	France	HCV patients with normal ALT (135)	3.6 years	Fibrosis, disease activity.
Miyakama et al (2009)[58]	Japan	Cirrhosis patients (132)	7.9 years	HCC.
Monica et al (2006)[59]	Italy	Elderly HCV patients (35)	10 years	HCC, liver mortality.
Nagao et al (2004)[60]	Japan	Community sample (509)	12 years	HCC, liver mortality.
Nakagawa et al (2009)[61]	Japan	HCV patients (333)	9 years	HCC.
Neal et al (2007)[62]	UK	HCV patients (2,285)	≥ 1 year	All cause & liver mortality.
Okuda & Yokosuka (2004)[63]	Japan	Renal patients (189)	> 4 years	Viral activity, cirrhosis.
Pergam et al (2008)[64]	US	Obstetric patients (2,528)	To delivery	Obstetric/maternal outcome.
Persico et al (2006)[65]	Italy	HCV patients (64)	10 years	Viral activity, cirrhosis.
Posthouwer et al (2007)[66]	Netherlands	Haemophilia patients (847)	≥15 years	ESLD.
*Pradat et al (2007)[67]	**8 Euro centres	HCV patients (962)	≤7 years	Cirrhosis, HCC, liver transplant, mortality
Prasad et al (2009)[68]	Switzerland	HCV patients (1,645)	2 years	All cause mortality
Punyagupta & Siwadune (2001)[69]	Thailand	Blood recipients (38)	≤24 years	Cirrhosis, HCC, mortality.
Rumi et al (2005)[70]	Italy	HCV patients (206)	5.9 years	Disease (ALT) activity.
Sangiovanni et al (2006)[71]	Sweden	HCV patients (214)	17 years	HCC, progression, mortality.
Sanyal et al (2006)[72]	US	HCV-cirrhosis/NASH Patients (302)	10 years	All cause & liver mortality.
Solá et al (2006)[73]	Spain	HCV/alcohol cirrhosis patients (377)	3.3 years	HCC, mortality & survival.
Strauss et al (2008)[74]	Sweden	HCV patients (36,126)	6.8 years	Primary liver Ca, HCC.
Tarao et al (2004)[75]	Japan	Cirrhosis patients (63)	>10 years	HCC.
Toshikuni et al (2009)[76]	Japan	HCV/alcohol cirrhosis	4.9 years	HCC, survival.

Authors (year)	Country	Population (n)	Follow up period	Outcomes measured
		Patients (227)		
Töz et al (2009)[77]	Turkey	Renal patients (197)	7.1 years	Progression, fibrosis.
Tsui et al (2007)[78]	US	Veterans (474,369)	4 years (retrospective)	HCV & ESRD.
Uto et al (2009)[79]	Japan	HCV patients (1,125)	8.2 years	All cause & liver mortality.
Wilson et al (2006)[80]	US	IDU (119)	4.2 years	Progression, fibrosis.
Wright et al (2003)[81]	**8 Euro centres	HCV patients (917)	11 years	Progression, cirrhosis.
Yawn et al (2002)[82]	US	HCV patients (355)	10 years (retrospective)	Referral to service patterns.
<u>Hepatitis C and B</u>				
Adachi et al (2008)[83]	Japan	Cirrhosis patients (123)	4.4 years	HCC.
Amin et al (2006)[84]	Australia	Case notifications (117,547)	12 years	HCC, other HC related Ca.
Duberg et al (2008)[85]	Sweden	Case notifications (45,353)	≤7.9 years	All cause & liver mortality.
Fujiwara et al (2008)[86]	Japan	BBV patients (70)	3.4 years	Fibrosis rate.
*Gannon et al (2009)[87]	US	BBV patients (10,263)	≤5.0 years	HCC, survival
Ikeda et al (2003)[88]	Japan	Cirrhosis patients (892)	7.5 years	HCC, survival and Ca treatment outcome.
*Ikeda et al (2007)[89]	Japan	BBV patients $HBV^{+/-}$ (846)	8.5 years	HCC.
Inoue et al (2009)[90]	Japan	Community sample (17,590)	12.7 years	HCC.
Mori et al (2000)[91]	Japan	Community sample (3,059)	4.8 years	HCC, viral/lifestyle factors.
Sasaki et al (2006)[92]	Japan	HCC post-surgery (417)	≤0.9 years	Survival, HCC recurrence
Sun et al (2003)[93]	Taiwan	Community sample males (12,008)	9.2 years	HCC.
Tanaka et al (2004)[94]	Japan	Blood donors (1,927)	8.3 years	HCC.
Tanaka et al (2006)[95]	Japan	HCV+ community sample (74)	12 years	HCC.
Wang et al (2007)[96]	Taiwan	Community sample (4,958)	7 years	Diabetes Type 2.
Zampino et al (2009)[97]	Italy	BBV patients (54)	23 years	HCC & cirrhosis.
<u>Hepatitis C and HIV</u>				
Anderson et al (2006)[98]	US	HIV patients (970)	4 years	Survival to AIDS, death.
Cheng et al (2007)[99]	US	HIV patients with Alcohol problems (396)	3.5 years	HCV impact on CD4 count
García-García et al (2006)[100]	Spain	Cirrhosis patients (1,217)	1.1 years	HCC.
Giordano et al (2004)[101]	US	HCV/ HIV patients (16,439)	10 years	HCC.
Grebely et al (2007)[102]	Canada	Community sample	6 years	HCV clearance, persistence

Authors (year)	Country	Population (n)	Follow up period	Outcomes measured
		(3,553)		
Hansen et al (2009)[103]	Denmark	HCV patients (1,780)	5 years (retrospective)	HCV treatment uptake.
Lumbreras et al (2006)[104]	Spain	IDU (3,247)	≤12 years	All cause mortality.
Merchante et al (2006)[105]	Spain	ESLD patients (153)	1.1 years	Decompensation, survival
Penida et al (2009)[106]	Spain	Cirrhosis patients (154)	3.0 years	Liver failure, liver mortality
Smit et al (2008)[107]	Netherlands	IDU (1,295)	≤11 years	Liver & all cause mortality.
Weis et al (2006)[108]	Denmark	HIV patients (2,734)	≤9 years	AIDS & all cause mortality.
Hepatitis C, B and HIV				
Bonacini et al (2004)[109]	US	HIV patients (472)	1.5 years	Liver mortality & HIV progression
Melendez-Morales et al (2007)[110]	US	Haemophilia patients (478)	3.0 years	HCV clearance inHIV, HBV

***** study also included in other study category
****** cohort drawn from 'HENCORE' clinical database
Abbreviations: ALT – alanine aminotransferase; **BBV – Blood Borne Virus (es); Ca** – cancer; **ESLD** – end stage liver disease; **ESRD** – end stage renal disease; **HCC** – hepatocellular carcinoma; **HCV** – hepatitis C virus; **HBV** – hepatitis B virus; **IDU** – injecting drug user; **NASH** – non alcoholic steatohepatitis

Table 4: Health related quality of life associated with antiviral treatment

Author (year)	Country	Study period (n)	Main measures	Outcomes
*Bonkovsky et al (2007)[111]	US	72 weeks (1,144) [all cirrhosis]	HR-QoL (SF-36)	Decreased QoL at baseline associated with histology severity & depression; improved post Tx if SVR.
*Dan et al (2006)[112]	US	72 weeks (271)	HR-QoL (SF-36)	Decreased SF-36 scores during Tx: associated with depression.
*Dan et al (2007)[113]	US	72 weeks (87)	HR-QoL (SF-36), anger (STAEI)	Increasing anger during Tx: associated with depression & ↓HR-QoL.
Evon et al (2009)[114]	US	72 weeks (114)	Depression (CES-D scale) social support (MOS)	12% depressed at baseline: most likely to exit early; low social support assoc-iated with new onset depression.
*Falasca et al (2009)[115]	Italy	Pre-post (18)	HR-QoL (SF-12), depression (BDI)	BDI unchanged, but SF-12 improved.
*Hollander et al (2002)[116]	Sweden	Pre-post (147)	HR-QoL (SF-36), Depression (CESD)	Baseline SF-36 reduced, reducing more in Tx, improving post Tx (best in SVR).
*McHutchison et al (2001)[117]	US	Pre-post (905)	HR-QoL (SF-36),	Baseline SF-36 reduced, improved in SVR (predicted by ALT, viral load, improved histology).
*Şahingöz et al (2009)[118]	Turkey	Pre-post (36)	HR-QoL (WHOQOL-BREF), anxiety (HARS), depression (HDRS)	High incidence of depression during IFN, associated with anxiety & reduced QoL

***** study also included in other study category
Abbreviations: BDI – Beck's Depression Inventory; **CESD** – Center for Epidemiological Studies Depression scale; **HARS** – Hamilton Anxiety Rating Scale; **HCV** – hepatitis C virus; **HBV** – hepatitis B virus; **HDRS** – Hamilton Depression Rating Scale; **IDU** – injection drug use; **NVR** – non viral response; **HR-QoL** – health related quality of life; **SF-36 (or 12)** – Short Form 36 (or 12); **STAEI** – State-Trait Anger Expression Inventory; **SVR** – sustained viral response; **Tx** – treatment; **WHOQOL-BREF** – World Health Organisation Quality Of Life assessment.

Table 5: Studies investigating the health outcomes after treatment.

Authors (year)	Country	Population (n)	Follow up period	Treatment outcome
Hepatitis C monoinfection:				
Bruggmen et al (2008)[119]	Switzerland	IDU/nonIDU (500)	0.5 years	SVR 64% in both.
*Bruno et al (2009)[44]	Italy	Cirrhosis (352)	14.4 years	Tx → did not improve clinical outcome
Chavalitdhamrong & Tanwandee (2006)[120]	Thailand	Post SVR (171)	3 years	No relapse, 1 HCC in baseline cirrhosis
Dalgard et al (2002)[121]	Norway	IDU/nonIDU post SVR (45)	5.3 years	No relapse, 1 IDU reinfected
Everhart et al (2009)[122]	US	Cirrhotic NVR (985)	3.8 years (retrospective)	50% long-term tx , increase progression in high BMI
Goodman et al (2009)[123]	US	NVR (535)	≤5 years	non-linear fibrosis (21-80%)
*Grebely et al (2009)[20]	Canada	High prevalence population (1,445)	5 years	1% tx uptake in HCV^+ →(SVR in 20%).
Ikeda et al (2005)[124]	Japan	Post SVR (1,056)	≥1 year	29 HCC (in med. 4.6 years)
*Ikeda et al (2009)[53]	Japan	Elderly (1,917)	6 years	HCC: Tx 11%; un-Tx 20%
Imazeki et al (2003)[125]	Japan	Treated/untreated (459)	8.2 years	SVR 80% fewer deaths.
Kobayashi et al (2007)[126]	Japan	Treated (1,124)	5.5 years	SVR 33%, HCC 6% (3.5% SVR)
Manesis et al (2009)[127]	Greece	Treated/untreated (1,738)	3.9 years (retrospective)	SVR 45%, HCC in 1.4% (lower in SVR)
Okanoue et al (2005)[128]	Japan	HCV (1,246)	7.7 years	13% HCC: ↑ in non- SVR
*Pradat et al (2007)[67]	**8 Euro centres	HCV (962)	≤7 years	SVR 25%, HCC 0.2 & death /transplant 0.4 /100 person years associated with NVR
Veldt et al (2007)[129]	Canada	Treated (479)	2.1 years	SVR 30%, 1/82 adverse outcomes in SVR
Co-infection				
De Bona et al (2007)[130]	Italy	HIV/HCV cirrhosis (50)	4.5 years	progression in NVR16% vs 52% un-treated
*Ikeda et al (2007)[89]	Japan	HBV/HCC	8.5 years	Tx response 41%: HCC only in HBV/HCV (11%)
Scott et al (2009)[131]	US	HIV/HCV (248)	4.4 years	Tx evaluation 44% (Tx 16%)
Zinkernagel et al (2006)[132]	Switzerland	HIV/HCV treated (160)	0.5 years	SVR 28:52% (type 1/4:2/3)
Liver transplant				
Ananthakrishnan & Saeian (2008)[133]	US	various liver diseases (13,855)	2 years	Lower survival in HCV
Bizollon et al (2005)[134]	France	SVR/NVR (80)	4.8 years	SVR → ↑ graft survival
*Feurer et al (2002)[135]	US	$HCV^{+/-}$ (75)	3 years	HCV recurrence in 10%, → ↓ functional performance
Gallegos-Orozco et al (2009)[136]	US	HCV	7 years (retrospective)	med. survival 3.5 years
*Gannon et al (2009)[87]	US	HBV & HCV (10,263)	≤5.0 years	HCC screen → ↑ survival post transplant

Authors (year)	Country	Population (n)	Follow up period	Treatment outcome
Hong et al (2008) [137]	Canada	$HCV^{+}/^{-}$ (1,842)	5 years	↓ 2 & 5 year survival in HCV
Selzner et al (2009)[138]	Canada	$HCV^{+}/^{-}$ (822)	5 years	↓ 2 & 5 year survival in HCV & increasing age
Takada et al (2006)[139]	Japan	HCV (91)	2.1 years	69% 5-yr survival in living donors
Verna et al (2009)[140]	US	$HCV^{+}/^{-}$ (709)	3.4 years	Biliary complications 22%; ↓1-year survival in HCV

***** study also included in other study category
****** cohort drawn from 'HENCORE' clinical database
Abbreviations: **ALT** – alanine aminotransferase; **Ca** – cancer; **ESLD** – end stage liver disease; **ESRD** – end stage renal disease; **HCC** – hepatocellular carcinoma; **HCV** – hepatitis C virus; **HBV** – hepatitis B virus; **IDU** – injection drug use; **NVR** – non viral response; **SVR** – sustained viral response; **Tx** - treatment

Table 6: Health related quality of life in untreated chronic hepatitis C infection.

Authors (year)	Country	Population (n)	Main QoL measures	Outcomes
Community and clinic populations:				
Bailey et al (2009)[141]	US	Clinic patients (135)	HR-QoL (adapted SF-36 & Cantrils Ladder); Depression (CESDS); illness uncertainty (MUIS)	Mod-high QoL, mild fatigue low depression - uncertainty main impact on QoL.
Balfour et al (2006)[142]	Italy	Clinic patients (123)	HR-QoL (SF-36); Depression (CESDC)	62% depressed; low QoL particularly in smokers.
Batista-Neves (2009) [143]	Brazil	Clinic patients (90)	HR-QoL (SF-36); Psych. diagnoses (MINI-Plus)	49% at least 1 psych. disorder; main association with reduced QoL
Coughlan et al (2002)[144]	Ireland	Female, medically acquired (93)	HR-QoL (SF-36, GHQ-30); mental health (HADS & SCAN)	High level psychological distress & reduced QoL not related to viraemia or liver histology.
Danoff et al (2006)[145]	US	Male, $HCV^{+}/^{-}$ (351)	HR-QoL (SF-36); depression (BDI); sexual function (BMSFI)	Reduced QoL in HCV - associated with sexual dysfunction (but not depression).
Gallegos-Orozco et al (2003)[146]	Mexico	Clinic patients (157)	HR-QoL (SF-36); depression (ZDS)	Reduced QoL in HCV, lower in low HCV knowledge.
*Gifford et al (2003)[147]	Australia	HCV^{+} females (462)	HR-QoL (SF-12)	Reduced QoL in HCV, relative to Australian norms.
*Gifford et al (2005)[148]	Australia	HCV^{+} males (308)	HR-QoL (SF-12)	Reduced QoL in HCV, relative to Australian norms.
Gunasekera et al (2008)[149]	Australia	Rural clinic patients (80)	HR-QoL (SF-36)	Reduced QoL in HCV, lower in Drug & Alcohol clinics (same as city HCV patients).
Häuser et al (2004)[150]	Germany	Clinic patients (88)	HR-QoL (SF-36, CLDQ), mental health (HADS)	Reduced HRQoL in HCV, associated with mental health & comorbidities (not viral infection).
Helbling et al (2008)[151]	Switzerland	Clinic patients (833)	HR-QoL (SF-36); Anxiety (HADS);	Reduced QoL in HCV, due to host & environment not viral factors.

Authors (year)	Country	Population (n)	Main QoL measures	Outcomes
Hickey et al (2008)[152]	Ireland	$HCV^{+}/^{-}$ females (236)	HR-QoL (SEIQoL-DW); Anxiety & depression (HADS); symptoms (IPQ)	More symptoms, anxiety & depression in medically acquired HCV – but no differences in QoL
Hussain et al (2001)[153]	US	Clinic patients (220)	HR-QoL (SF-36)	Reduced QoL in HCV, ALT or drug & alcohol not associated.
Kallman et al (2006)[154]	US	$HCV^{+}/^{-}$ (191)	HR-QoL (SF-36, CLDQ); fatigue (CFS)	Very reduced QoL in HCV, fatigue most important contributer to ↓ QoL.
*Kang et al (2005)[155]	Taiwan	$HCV^{+}/^{-}$ (560)	HR-QoL (SF-36)	Reduced QoL in HCV.
Kramer et al (2002)[156]	Austria	Clinic patients (100)	HR-QoL (SF-36); fatigue (FIS) & neuro-cognitive function	Significantly reduced QoL relative to population norms. Slight but significant neuro-cognitive impairment in HCV not associated with disease activity, fatigue or QoL
Kramer et al (2005)[157]	Austria	Clinic patients (120)	HR-QoL (SF-36); fatigue (FIS) & neuro-cognitive function	Significant fatigue in HCV independently associated with reduced QoL, but not with brain dysfunction
Kwan et al (2006)[158]	US	Male, $HCV^{+}/^{-}$ (3,023)	HR-QoL (SF-36)	Reduced QoL in HCV, independent of medical/psych. comorbidities.
Lim et al (2006)[159]	US	Male, $HCV^{+}/^{-}$ (3,023)	HR-QoL (SF-36)	Reduced QoL in HCV, predicted by depression & post-traumatic stress.
Mikocka-Walus et al (2008)[160]	Australia	$HCV^{+}/^{-}$ gastro. patients (139)	HR-QoL (SF-12); anxiety & depression (HADS); psychology (SCL090)	Reduced QoL in all patients, higher prevalence depression and comorbid psychological conditions in HCV
Miller et al (2001)[161]	Australia	Clinic patients (95)	HR-QoL (SF-36); social support (SSM)	Reduced QoL in HCV, ALT & social support not related, social stigma common.
Moyer et al (2007)[162]	Canada	Clinic patients (123)	HR-QoL (SF-36); comorbidities (BSI)	Reduced QoL in HCV, lower in pessimistic persons.
Pojoga et al (2004)[163]	Italy	$HCV^{+}/^{-}$ (102)	HR-QoL (adapted SF-36)	Reduced QoL in HCV, ALT not related.
Rowan et al (2005)[164]	US	Clinic patients (62)	HR-QoL (SF-36, CLDQ); psychosocial (SCID-CV; BDI; BAI; abbreviated CM; SSM	Reduced QoL in HCV, not related to clinical/viral/substance use/psych. disorder.
Schwarzinger et al (2004)[165]	Egypt	$HCV^{+}/^{-}$ (1,282)	HR-QoL (SF-12)	No difference in QoL or ALT – not aware of HCV status.
Teixeira et al (2006)[166]	Brazil	Blood donors (120)	HR-QoL (SF-12)	Reduced QoL in for all stages HCV, not related to lifestyle/demographic factor except female sex.
Teuber et al (2008)[167]	Germany	Clinic patients (123)	HR-QoL (SF-36);	Reduced QoL associated with

Authors (year)	Country	Population (n)	Main QoL measures	Outcomes
			fatigue (FIS)	fatigue and fibrosis.
von Wagner et al (2009)[168]	Germany	$HCV^{+}/^{-}$ (165)	HR-QoL (ELQ); psycho-emotional health (PMS)	Reduced QoL in HCV, inc-reased anger & depression, not related to ALT.
Former or current IDU:				
Dalgard et al (2004)[169]	Norway	Current IDU (199)	HR-QoL (SF-36)	Reduced QoL associated with HCV status awareness, heroin use, female sex.
Gjeruldsen et al (2006)[170]	Norway	HCV^{+}/non IDU-HCV^{-} (68)	HR-QoL (SF-36)	Reduced QoL in HCV, relat-ed to drug use & marital status.
Viral co-infections:				
Baum et al (2008)[171]	US	HIV mono- & co-infected drug users (192)	HR-QoL (SQLI)	Reduced QoL in HCV-HIV co-infection, related to dep-ression, ↑symptoms, ↑ health care utilisation, low HCV Tx access.
Braitstein (2005)[172]	Canada	HIV mono & co-infected (484)	HR-QoL (MOS-SF); mental health (CESD)	Reduced QoL in HCV-HIV most due to drug use & SE factors.
Fleming et al (2004)[173]	US	HIV & HCV mono- & co-infected (299)	HR-QoL (HQLQ)	Reduced QoL but similar across groups, predicted by IDU, psych illness,unemploy-ment.
Ozkan et al (2006)[174]	Turkey	HBV or HCV (107)	HR-QoL (SF-36); mental health (SCID-CV)	Reduced QoL in HCV & HBV, psych. morbidity associated.
Svirtlih et al (2008)[175]	Serbia	Inpatient HBV or HCV (227)	HR-QoL (SF-12)	Reduced QoL in HCV & HBV, cirrhosis associated.
Thein et al (2007)[176]	Australia	HIV & HCV mono- & co-/un-infected (94)	HR-QoL (HQLQ); mood (DASS); cognit-ive function (NART, TMT)	Little differences btwn HCV & HIV/HCV, but some in HIV/HCV vs controls.
Tsui et al (2007) [177]	US	HIV^{+} homeless (216)	HR-QoL (SF-36)	Reduced QoL overall, lower physical health scores in HCV/HIV co-infection
Various stages of disease progression:				
Björnsson et al (2009)[178]	Sweden	$HCV^{+}/^{-}$ (472)	HR-QoL (SF-36, EQ_{index})	Impaired QoL in HCV, relat-ed to liver disease severity.
Chong et al (2003)[179]	Canada	HCV patients (193)	HR-QoL (SF-36, $EQ_{index,}$ HUI)	Reduced QoL in HCV, small differences related to disease severity (ALT not related).
Córdoba et al (2003)[180]	Spain	HCV patients (120)	HR-QoL (SF-36); mental health (BDI,STAI) neuropsychology (AVL)	Reduced QoL unrelated to cognitive function, lower in severe disease (beta blockers, diuretics implicated).
Hsu et al (2009)[181]	Canada	HCV patients (271)	HR-QoL (HQLQ, HUI, TTO)	Reduced QoL partially due to to disease severity, QoL ass-ociated with comorbidity & SE and marital status.

Authors (year)	Country	Population (n)	Main QoL measures	Outcomes
Comorbidities:				
Asfar et al (2009a)[182]	Turkey	$HCV^{+}/^{-}$ haemodialysis patients (165)	HR-QoL (SF-36); depression (BDI)	Reduced QoL in HCV^{+} dialysis patients vs HCV^{-}
Asfar et al (2009b)[183]	Turkey	HCV^{+} haemodialysis patients with HCV (32)	HR-QoL (SF-36); depression (BDI)	QoL not related to liver, but lower in depression, anaem-ia, malnutrition.
Posthouwer et al (2005)[184]	Netherlands	Haemophilia (602)	HR-QoL (SF-36)	Reduced QoL in HCV, independent of age, HIV, severity of haemophilia.
Marcellin et al (2007)[185]	France	115 HIV-HCV coinfect-ed patients	HR-QoL (WHOQOL-HIV Bref), depression (CES-D), fatigue (FIS)	Fatigue and depression acc-ount for the majority of var-iance in QoL scores noted. Fatigue associated with imp-aired physical QoL and social relationships, depression ass-ociated with lower environ-mental QoL (eg satisfaction with home, transport, health care access and quality, etc)

* study also included in other study category

Abbreviations: **ALT** – alanine aminotransferase; **AVL** – Auditory Verbal Learning; **BAI** – Beck Anxiety Inventory; **BDI** – Beck Depression Inventory; **BMSFI** – Brief Male Sexual Function Inventory; **BSI** – Brief Symptoms Index; **CESD** - Center for Epidemiological Studies Depression scale; **CFS** – Chronic Fatigue Screener; **CLDQ** – Chronic Liver Disease Questionnaire; **CM** – Cook Medley anger measure; **DASS** – Depression Anxiety Stress Scales; **EFQ** – Everyday Life Questionnaire (related to SF-36); $\mathbf{EQ_{index}}$ – European Quality of life index; **FIS** – Fatigue Impact Scale; **HADS** – Hospital Anxiety and Depression Scale; **HCV** – hepatitis C virus; **HQLQ** – Hepatitis Quality of Life Questionnaire (incorporating the SR-36); **HUI** – Health Utilities Index; **IDU** – injection drug use; **IPQ** – Illness Perception Questionnaire; MINI-**Plus** – Mini International Neuropsychiatric Interview; **MUIS** – Mishel Uncertainty in Illness Scale; **NART** – National Adult Reading Test; **PMS** – Profile of Mood States; **QoL** – quality of life; **SCAN** – Schedule for Assessment in Neuropsychiatry; **SCL-90** – Symptom Checklist-90-R; **SSM** – Medical Outcomes Study Social Support Measure; **SCID-CV** – Structured Clinical Interview for DSM-IV Axis I Disorders; **SEIQoL-DW** – Schedule for the Evaluation of Individual Quality of Life-Direct Weighting; **SQLI** – Spitzer Quality of Life Index; **STAI** – State Trait Anxiety Inventory; **TMT** – Trail Making Tests; **TTO** – Time Trade-Off instrument; **Tx** – treatment; **WHOQOL-HIV Bref** – World Health Organization Quality of Life –HIV Bref Questionnaire; **ZDS** – Zung Depression Scale

Table 7: Health related quality of life during and after treatment/transplant for hepatitis C infection.

Authors (year)	Country	Population (n)	Main QoL measures	Outcomes
During antiviral treatment:				
Batki et al (2009)[186]	Taiwan	On treatment (115)	HR-QoL (HQLQ); Social support (ISSB)	Low QoL, associated with finance, long Tx. duration & low social support.
Bianchi et al (2000)[187]	Italy	On treatment/not (126)	HR-QoL (SF-36, NHP); Depression (CESD); Psych. diagnoses (MINI-Plus)	Reduced QoL in HCV, further reduces by Tx.
Chang et al (2008)[188]	Taiwan	On treatment (115)	HR-QoL (HQLQ); social support (ISSB)	Reduced QoL associated with depression and anger during treatment.
*Bonkovsky et al (2007)[111]	US	On/end treatment (1,144) [all cirrhosis]	HR-QoL (SF-36)	Decreased QoL at baseline associated with histology severity & depression; improved post Tx if SVR.

Authors (year)	Country	Population (n)	Main QoL measures	Outcomes
*Dan et al (2006)[112]	US	On/end treatment (271)	HR-QoL (SF-36)	Decreased SF-36 scores during Tx: associated with depression.
*Dan et al (2007) [113]	US	On/end treatment (87)	HR-QoL (SF-36), anger (STAEI)	Increasing anger during Tx: associated with depression & ↓HR-QoL.
Fontana et al (2002)[189]	US	On treatment/not (126)	HR-QoL (SF-36), symptoms (BSI)	Reduced QoL associated with emotional distress, particularly in untreated & those expecting fatal outcome.
*Kang et al (2005)[155]	Taiwan	On treatment (47)	HR-QoL (SF-36)	Reduced QoL in Tx, lower by end Tx, no improvement in NVR (improved in SVR)
Hilsabeck et al (2005)[190]	US	Pre (30) & post treatment (11)	HR-QoL (SF-36) Depression/anxiety (BDI-II, BAI), cognitive function (SDMT &TMT)	Treatment associated with reduced cognitive abilities. QoL at baseline did not differ between Tx groups. QoL impact of Tx not reported.
*Hollander et al (2002)[116]	Sweden	On/end treatment (147)	HR-QoL (SF-36), depression (CESD)	Baseline SF-36 reduced, reducing more in Tx, improving post Tx (best in SVR).
Hopwood & Treloar (2005)[191]	Australia	Treatment experience (19)	In-depth semi-structured interviews[qualitative study]	All reported side-effects → ↓QoL; describes subjective negative responses and impact on social interaction; individual response & coping.
*McHutchison et al (2001)[117]	US	On/end treatment (905)	HR-QoL (SF-36),	Baseline SF-36 reduced, improved in SVR (predicted by ALT, viral load, improveed histology).
*Şahingöz et al (2009)[118]	Turkey	On/end of treatment (36)	HR-QoL (WHOQOL-BREF), anxiety (HARS), depression (HDRS)	High incidence of depression during IFN, associated with anxiety & reduced QoL
Schäfer et al (2009)[192]	Germany	$HCV^{+/-}$ ORT patients on treatment /not (2,414)	HR-QoL (WHO Euro-QOL EQ-5D), mental health (BSI)	Reduced QoL and sleep disturbance in HCV, but IFN not related to increased depression/reduced QoL/reduced ORT efficacy.
<u>After antiviral treatment:</u>				
Akyüz et al (2009)[193]	Turkey	Treated/untreated HCV^{+} renal failure patients (95)	HR-QoL (SF-36), depression (BDI)	Low QoL with little differences between groups, IFN → reduced QoL further,general health perception improved in SVR.
*Falasca et al (2009)[115]	Italy	End of treatment (18)	HR-QoL (SF-12), depression (BDI)	Improved subjective QoL, depression levels unchanged.
Fontana et al (2001)[194]	US	Unsuccessful Tx (107)	HR-QoL (HQLQ, HUI, TTO);	Reduced QoL, not related to ALT, alcohol, drugs or other demographic/viral factors. ↓QoL associated with comorbidities.
John-Baptiste et al (2009)[195]	Canada	SVR/NVR (235)	HR-QoL (HQLQ,	Reduced QoL in NVR, imp-

Authors (year)	Country	Population (n)	Main QoL measures	Outcomes
			HUI, TTO); comorbid-dities (ICED)	roved QoL in SVR & benefit sustained over time.
Taliani et al (2007)[196]	Italy	Untreated/relapsers/ non-responders (264)	HR-QoL (SF-36, CLDQ, WHOQOL-BREF)	Reduced QoL in all groups, lowest in NR, associated with physical comorbidity.
After liver transplantation:				
Dudley et al (2007)[197]	UK	Post transplant (8)	Indepth interviews [qualitative study]	Transplant replaces one health state with other; feelings of stigma & future uncertainty may be worse in HCV transplants.
*Feurer et al (2002)[135]	US	$HCV^{+}/^{-}$ post transplant (75)	HR-QoL (SF-36), psychosocial (PAI)	HCV recurrence in 10%: associated with ↓functional performance →↓ QoL.
Paterson et al (2000)[198]	US	Post transplant (87)	HR-QoL (SF-36), depression (BDI), adaption (BMICIS) psycho-emotional stress (POMS)	HCV recurrence in 62%: associated with ↓QoL and ↓functional performance (no difference in adaptive coping)

* study also included in other study category

Abbreviations: **ALT** – alanine aminotransferase; **BAI** – Beck Anxiety Inventory; **BDI** – Beck Depression Inventory; **BMICIS** – Billing and Moos Inventory of Coping with Illness Styles; **BMSFI** – Brief Male Sexual Function Inventory; **BSI** – Brief Symptoms Index; **CESD** - Center for Epidemiological Studies Depression scale; **CLDQ** – Chronic Liver Disease Questionnaire; **HARS** – Hamilton Anxiety Rating Scale; **HCV** – hepatitis C virus; **HDRS** – Hamilton Depression Rating Scale; **HQLQ** – Hepatitis Quality of Life Questionnaire (incorporating the SR-36); **HR-QoL** – health related quality of life; **HUI** – Health Utilities Index; **ICED** – Index of Co-Existent Disease; **IFN** – interferon; **ISSB** – Inventory of Socially Supportive Behaviours; **MINI-Plus** – Mini International Neuropsychiatric Interview; **NHP** – Nottingham Hill Profile; **NR** – non responders; **ORT** – opiate replacement therapy; **PAI** – Psychosocial Adjustment to Illness scale; **POMS** – Profile Of Mood States scale; **QoL** – quality of life; **SDMT** – Symbol Digit Modalities Test); **SVR** – sustained viral response; **TMT** – Trail Making Tests; **TTO** – Time Trade-Off instrument; **Tx** – treatment; **WHOQOL-BREF** – World Health Organisation Quality Of Life assessment.

Table 8: Psychosocial experience of living with hepatitis C infection.

Authors (year)	Country	Participants	Methodological approach	Outcomes and conclusions
Social functioning:				
Blasiole et al (2006)[199]	US	352 HCV clinic patients	Primarily quantitative ; with semi-structured interviews.	Low social support, associat-ed with living alone, unem-ployment, IDU history, anx-iety & depression, and nega-tive mood state
Carrier et al (2005)[200]	Canada	36 $HCV^{+}/^{-}$ IDU	Qualitative study invol-ving individual interviews and ethnographic observ-ation	HCV given meaning by soc-ial interactions, media repre-sentations & person experie-ces. Social implications (↑stigma) → high anxiety.
Grundy & Beeching (2004)[201]	UK	8 female HCV clinic patients	Qualitative study invol-semi-structured inter-views	Fear of transmission impacts on family & sexual relation-ship→ concerns about ability to fulfil gender roles.
Janke et al (2008) [202]	US	40 HCV clinic patients	Qualitative study invol-	Intense anger and ↓impulse

Authors (year)	Country	Participants	Methodological approach	Outcomes and conclusions
			ving focus groups	control; transmission fears→ self-imposed isolation → ↓social support (even in Tx).
Sgorbini et al (2009)[203]	Australia	5 HCV patients on Tx & their partners	Qualitative study involving individual semi-structured interviews	IFN substantial QoL impact; 4 themes – illness & Tx (↓ health, isolation), keeping a secret (disclosure, stigma), sharing experience (lending a hand, parenting in adversity), enduring struggle (finance, future etc).
Temple-Smith et al (2004)[204]	Australia	32 HCV^+ people	Qualitative study involving in-depth interviews	Gender related differences in symptom recognition, health seeking behaviour and notions of social support (males likely to dismiss HCV impact & education + support needs.)
<u>Lifestyle:</u>				
Castera et al (2006b)[205]	France	40 HCV clinic patients	Quantitative study, involving surveys, demographic & clinical data.	59% thought HCV always fatal; Sex-life changes in 58% (associated with males, perceived disease severity, anxiety) ; dietary changes in 48% (associated with age, anxiety).
Roy et al (2007)[206]	Canada	39 HCV^+ IDU	Qualitative study involving in-depth interviews	HCV 'trivial' in context of other priorities, and in relation to HIV. But did aim to minimise transmission.
Scognamiglio et al (2007)[207]	Italy	27 HCV clinic patients	Quantitative study, involving surveys, demographic & lifestyle data	75% drinkers modified alcohol intake; 21% smokers modfied smoking; and 64% modified diet after diagnosis.
Sokyan et al (2005)[208]	Turkey	46 HCV clinic patients	Quantitative study, involving surveys, demographic, clinical & lifestyle data	Sexual dysfunction in 35%: predicted by depression (and GGT levels).
Wright et al (2005)[209]	UK	17 HCV^+ homeless IDU	Qualitative study involving in-depth interviews	Ongoing risk practices with some adoption of harm reduction (cleaning needles, washing spoons); modified diet, reduced drinking since diagnosis.
Zacks et al (2006)[210]	US	175 HCV clinic patients	Quantitative study, involving surveys, demographic, social functioning data	Less likely to share s towels, or drinking glass or prepare food. 20% less kissing, 27% less intercourse; majority reported financial difficulty, internalised shame, social rejection.

Authors (year)	Country	Participants	Methodological approach	Outcomes and conclusions
Health and Wellbeing:				
Bova et al (2008)[211]	US	39 HIV-HCV coinfected patients	Mixed method study: involving surveys incorporating HIV-SEI and MHI-5; semi-structured interviews	Average of 10 symptoms: pain fatigue GI problems most 'both-ersome'. 33% had poor mental well-being associated with greater symptom burden. Difficulty differentiating HCV and HIV symptoms. Symptom burden of coinfected might not be greater than HIV alone.
Conrad et al (2006)[212]	Australia	32 HCV^{+} people	Qualitative study involving semi-structured interviews	Debilitating "hep C attacks" associated with depression & views of HCV as fatal; fear of transmission &uncertainty common; universal anxiety about stigma & discrimination.
Copeland et al (2004)[213]	Scotland	16 HCV^{+} ID	Qualitative study involving semi-structured interviews	Explored themes that explained circumstances leading to 'problem drug user' identity: problems growing up, ↑unemployment & ↓education, peer influence in IDU behaviour.
Dwight et al (2000)[214]	US	50 HCV patients	Quantitative study involving structured psychiatric interviews and validated questionnaires including MAF-FQ & SF-36	28% had depressive disorders not correlated with disease severity or demographics. Severity of depression correlated with fatigue level. Concludes disability and fatigue more related to depression than hepatic disease.
Forton et al (2002)[215]	UK	27 $HCV\text{-}RNA^{+}$ & 16 HCV-cleared patients	Quantitative study, involving cognitive assessment; QoL, fatigue & depression questionnaires	RNA+ impaired on more cognitive tasks. Impairment not accounted for by depression, fatigue, or IDU history. Suggests biological cause
Glacken et al (2001)[216]	Ireland	9 HCV^{+} people	Qualitative study involving in-depth interviews	None experiencing health transition following diagnosis: impeded by uncertainty, ↓knowledge, 'contamination'; Facilitated by social support, information community participation, being positive.
Glacken et al (2003)[217]	Ireland	28 HCV^{+} people	Qualitative study involving in-depth interviews particularly focusing on fatigue	Fatigue is multidimensional with 2 distinct types - chronic and idiopathic. Universally experienced as 'whole-body-sensation'. Suggests an HCV cause of fatigue that is perpetuated by secondary factors such as inactivity, pain and disability
Golden et al (2005)[218]	Ireland	90 HCV clinic patients	Quantitative study, involving structured psychiatric interviews and validated questionnaires	High prevalence of undiagnosed mood disorders. Depression associat ed with adverse experiences of illness (including

Authors (year)	Country	Participants	Methodological approach	Outcomes and conclusions
			including SCID-CV	stigma,poor adjustment & physical symptoms), anxiety not associated with any risk factor.
Goulding et al (2001)[219]	Ireland	77 HCV clinic patients	Quantitative study, involving structured clincal examination & history FIQ and HADS	Moderate increase in fibromyalgia in HCV with number of tender points associated with mode of acquisition. Anxiety & depression higher in HCV, possibly related to psychological factors.
Grassi et al (2002)[220]	US	290 $HCV^{+}/^{-}$ & $HIV^{+}/^{-}$ IDU	Quantitative study, involving surveys, demographic, psychosocial data	Higher OCD, paranoid ideation, phobic anxiety in HCV (v HIV); lower 'fighting spirit', higher hopelessness & anxious disease preoccupation in HCV (v HIV).
Groessl et al (2008)[221]	US	8 male HCV clinic patients	Qualitative study involving semi-structured interviews	Pronounced social impact of diagnosis; greater impact in non-IDU; psychological issues prevalent; barriers to treatment and care common.
Hassoun et al (2002)[222]	Canada	92 HCV patients	Quantitative study, involving demographic clinical data, assessing fatigue with the FIS	67% had fatigue – described as 'frequent and disabling'. Significantly increased compared to controls, fatigue severity not associated with disease activity or progress.
Hilsabeck et al (2002)[223]	US	66 HCV^{+} &14 HCV- patients with liver disease	Quantitative study, involving assessment of four cognitive functions using validated cognitive tests	Significant cognitive difficulties for both patient groups. associated with liver fibrosis but still higher in HCV with and without cirrhosis.
Hilsabeck et al (2003)[224]	US	21 HCV patients	Quantitative study, involving assessment of four cognitive functions using validated cognitive tests	Significant cognitive difficulties not related to psychiatric symptoms. Cognitive impairment level sufficient to impact on QoL.
Hogg et al (2003)[225]	Canada	241 transfusion associated HCV patients & 222 HCV^{-} patients	Quantitative study, involving surveys, demographic & clinical data	HCV more likely to have ≥ 2 symptoms, have worse health status than 10 years ago , and have higher illness intrusiveness rating.
Hopwood & Treloar (2008)[226]	Australia	20 patients on/recently HCV treatment	Qualitative study involving semi-structured, in-depth interviews	Past experiences of marginalisation (disadvantage; other chronic illnesses, etc) →enhanced resilience in coping with HCV Tx.
Kinder (2009)[227]	US	8 males, IFN treated	Qualitative study invol- In depth, semi-structured Interviews	Confusion & surprise re diagnosis; anxiety re treatment decisions related to HCV seriousness & fear of side-effects; tx e xperience was negative with significant physical, emotional

Authors (year)	Country	Participants	Methodological approach	Outcomes and conclusions
				side-effects and concern re paradox of injecting IFN.
Kozanoglu et al (2003)[228]	Turkey	95 HCV^+ patients & 95 HCV-	Quantitative study of demographic, clinical data, information on fibromyalgia	Higher prevalence of fibromy-algia syndrome (FS) in HCV with more tender spots with higher pain intensity compar-ed to controls with FS
Kraus et al (2000)[229]	Germany	Post transplant (87)	Quantitative study, involving surveys, demo-graphic & clinical data	Depression in 22%, anxiety 15%: associated with age, ad-vice not to seek Tx, recent di-agnosis. Emotional state or coping style not associated with infection mode or liver damage
Lang et al (2006)[230]	Australia	Phase 1- 73 people with HCV; Phase 2- 188 people with HCV	Phase 1 – focus groups & individual interviews Phase 2 – quantitative surveys and serology	Tiredness, irritability, depress-ion, mental tiredness & abdom-inal pain most prevalent symp-toms. 4 symptom 'clusters': neuropsychiatric; gastro-intest-inal; algesic (e.g. joint & musc-le pain); and dysthetic (eg noise/light sensitivity, skin problems)
Lehman et al (2002)[231]	US	120 veterans with HCV	Quantitative study, involving surveys, demo-graphic & clinical data	Depression in 44%, anxiety in 38%, post traumatic stress dis-order 21%; alcohol-related problems 27%. All outcomes positively correlated with each other.
McAndrews et al (2005)[232]	Canada	37 HCV patients & 46 HCV- controls	Quantitative study involving radiographic, clinical and neuropsych-ologic assessment	Marginally reduced learning efficiency in HCV not associat-ed with increased fatigue and depression in this group. HCV group had changes in brain metabolites, not associated with liver histology and not explan-atory of 'brain fog' reported by people with HCV
McDonald et al (2002)[233]	Australia	115 HCV clinic patients	Quantitative study, involving surveys, demo-graphic & clinical data fatigue (FIS) symptoms (modified SCL-90-R)	Fatigue in 83%, all the time in 31%, psychopathology in 50% Fatigue weakly correlated with liver histology but strongly correlated with all psycholog-ical domains, particularly depression
McKenna et al (2009)[234]	Ireland	290 people with iatro-genically acquired HCV	Quantitative survey of sociodemographic and clinical characteristics	After mean 26 years infection 62% hepatic & 99% extra-hepatic symptoms. Fatigue in 88% and pain in 96%. 89% reported diagnosed co-morbid diseases. Reported symptoms associated with age, female sex, and co-morbidities
Nagano et al (2004)[235]	Japan	69 HCV patients	Quantitative study in-volving clinical and demographic data,	All stress inventory scales rel-ated to the 'type 1' personality (low sense of control, objective

Authors (year)	Country	Participants	Methodological approach	Outcomes and conclusions
			'stress inventory'	dependence of loss, unfulfilled need for acceptance, altruism) associated with severity of disease (according to ALT and liver cirrhosis). Chronic stress related to type 1 personalities might influence HCV progression
Obhrai, et al (2001)	US	149 HCV+/- patients with/without alcohol problems and/or liver disease	Quantitative study, involving surveys, demographic & clinical data	Fatigue higher in HCV cf other groups (not statistical) but did not improve with rest as effectively. Fatigue and psychological symptoms more common, severe and less responsive to remedies in HCV
Piche et al (2002)[236]	France	78 HCV patients, 22 HCV- controls, 13 billiary cirrhosis patients	Quantitative study, involving surveys (FIS), demographic & clinical data	Fatigue higher in HCV and more in females. Correlated with leptin (as in billiary cirrhosis) but not age, liver function, or viral load
Poynard et al (2002)[237]	France	1614 HCV patients	Quantitative study, retrospective analysis of demographic, clinical, virologic, immunologic and other data	Fatigue in 53%, severe enough to impair activity in 17%, independently associated with female sex, age > 50 years, cirrhosis, depression and purpura
Silberbogen et al (2007)[238]	US	29 veterans with HCV (32% on Tx)	Quantitative study, involving data on pain (MPQ-SF; WHYMPI; NRS) & depression (CES-D)	Pain reported in 83%, most in upper limb, hip, lower limb & lower back. Alcohol and smoking associated with pain and depression in HCV.
Weissenborn et al (2004)[239]	Germany	30 HCV patients with fatigue, 15 HCV- controls	Quantitative study, involving neurological & neuropsychological assessment, EEG, MRI & MRS	Some cognitive impairment, higher anxiety and depression (none had cirrhosis or fibrosis) Evidence of central nervous system involvement (per MRI), associated with higher severity of fatigue
Yovtcheva et al (2001)[240]	US	306 veterans with HCV	Quantitative study, retrospective analysis of past & present DSM-IV based psychiatric disorders recorded in clinical database	Mood disorders in 38%, personality disorders in 30%, post traumatic stress disorder in19% and other in 17%. 86% diagnosed with alcohol disorders & 28% with IDU history (noted high prevalence of psychiatric disorders exist in veterans)

* study also included in other study category

Abbreviations: **BDI-II** – Becks Depression Inventory-II; **BAI** – Becks Anxiety Inventory; **CES-D** – Center for Epidemiologic Studies Depression Scale; **EEG** – electroencephalography; **FIQ** – Fibromyalgia Impact Questionnaire; **FIS** – Fatigue Impact Scale; **FSS** – Fatigue Severity Scale; **HADS** – Hospital Anxiety and Depression Score; **HCV** – hepatitis C virus; **HIV-SEI** – HIV Symptom Experience inventory; **IDU** – injection drug user; **MAF-FQ** – MAF Fatigue Questionnaire; **MHI-5** – Mental Health Index-5; **MPQ-SF** – McGill Pain Questionnaire-Short Form; **MRI** – magnetic resonance imaging; **MRS** – magnetic resonance spectroscopy; **NRS** – Numeric Rating Scale; **SCID-CV** – Structured Clinical Interview for DSM-IV Axis I Disorders: Clinician Version; **SCL-90-R** – Symptom Checklist, 90 item; **SF-36** – Short Form 36; **QoL** – quality of life; **Tx** – treatment; **WHYMPI** – West Haven-Yale Multidimensional Pain Inventory

Table 9: Diagnosis, management and treatment of chronic hepatitis C infection.

Authors (year)	Country	Participants	Methodological approach	Outcomes and conclusions
<u>Diagnosis impact</u>				
Castera et al (2006a)[241]	France	185 HCV clinic patients	Quantitative study, involving surveys, demographic & clinical data	Diagnosis a major stressful event: < death of loved one or divorce, but > home removal or job loss. Associated with perceived disease severity.
Craine et al (2004)[242]	UK	43 HCV$^{+/-}$ IDU	Qualitative study involving semi-structured interviews and focus groups	Lower risk behaviour in HCV$^-$ variable risk behaviour after HCV diagnosis (availability) of clean injecting equipment greater influence).
Gill et al (2005)[243]	Pakistan	90 recently diagnosed HCV patients	Quantitative study, involving surveys, demographic, anxiety & clinical	Diagnosis related stress associated with perceived disease severity: > divorce, loss of income source, or moving to another city.
Harris et al (2009)[244]	Australia & New Zealand	40 HCV$^+$ people	Qualitative study involving semi-structured interviews	HCV diagnosis: 'no big deal' 50%, 'shocking' 'devastating' 50%. Meaning of HCV varied with social context; 'calculable risk' – better than HIV. HCV more stigmatising than HIV in co-infected; other competing life issues a higher priority.
Sutton & Treloar (2007)[245]	Australia	36 HCV$^+$ people	Qualitative study involving semi-structured interviews	HCV shifted in priority when competing with other pressures - weighed against dynamic social and health circumstance.
Tomkins et al (2005)[246]	UK	17 HCV$^+$ homeless IDU	Qualitative study involving in-depth interviews	Diagnosis → shock, anger, devastation + long-lasting emotional psychosocial physical effects. Stigma and unpredicatable effects of disclosure. (↑info→ ↓negative diagnosis impact.
<u>Stigma and discrimination:</u>				
Crockett et al (2004)[247]	Australia	17 HCV$^+$ IDU	Qualitative study involving semi-structured interviews	Social exclusion & marginalisation; profound impact of stigma (feeling 'unclean' etc) related to transmission fears; perceived discrimination in health care; child-bearing decisions affected by transmission fears.
Fraser & Treloar (2006)[248]	Australia	6 HCV$^+$ people	Qualitative study involving semi-structured interviews	Stigmatising responses, sense of contamination, inevitability of disclosure. Despair→ belief infection with other BBV not important.

Authors (year)	Country	Participants	Methodological approach	Outcomes and conclusions
Golden et al (2006)[249]	Ireland	87 HCV clinic patients	Quantitative study, involving surveys, demographic, psychiatric, stigma &clinic data	Fear of disclosure; social isolation & social rejection; stigma associated with depression & ↓SE status; stigma associated with ↓illness acceptance, social adjustment and ↑symptoms.
Hopwood et al (2006)[250]	Australia	504 HCV^+ people	Quantitative study, involving surveys, demographic, social & risk factor data	Health care discrimination in 65%; negative health impact of discrimination in 45%: discrimination associated with pessimism re future health, ↓social interaction, HCV fatigue, knowing ↑people with HCV.
Zickmund et al (2003)[251]	US	257 HCV clinic patients	Mixed methods involving surveys and semi-structured interviews	HCV stigma in 57%: associated with females; loss of control; anxiety & depression; ↓QoL (no other demographic factor); Stigmatised reported problems in healthcare, work, families.
Health service access:				
Banwell et al (2005)[252]	Australia	462 HCV^+ females	Quantitative study, involving surveys, demographic, psychosocial data	Lesbians more dissatisfied with GP; heterosexuals more likely to be referred to specialist; lesbians more likely to use alternative health services. HCV experience of health services differs per sexual identity.
Bratstein et al (2006)[253]	Canada	707 HCV $^+$- $HIV^{+/-}$ IDU	Quantitative study, involving surveys, demographic,clinical & management history data	HCV-mono reported highest rate HCV symptoms, but HCV-HIV coinfected had more HCV health care utilisation
Butt et al (2008)[254]	Canada	26 HCV clinic patients	Qualitative study involving open interviews & daily participant recordings.	All described stigma in many settings: associated with fear of transmission & IDU association. Stigma = barrier to health care access (reports of refusal of care).
*Gifford et al (2003)[147]	Australia	462 HCV^+ females	Quantitative study, involving survey (SF-12) & social & clinical data	52% referred to specialist but Tx only in 17%; low health status and 50% report symptoms; 48% report less favourable treatment from health professionals. (high need but low specialist service access, particularly in IDU)
*Gifford et al (2005)[148]	Australia	308 HCV^+ males	Quantitative study, involving survey (SF-12) & social & clinical data	30% referred to specialist but Tx only in 20%; low health status and 58% report symptoms; 40% report less favourable treatment from health professionals. (high need but low specialist service access).
Harris et al (2005)[255]	New Zealand	20 HCV clinic patients	Qualitative study involving individual	Frustration with reduced info from doctors; dissatisfied with

Authors (year)	Country	Participants	Methodological approach	Outcomes and conclusions
			interviews	liver focus (only IFN on offer); many told diagnosis on phone; common reports withdrawal/refusal health treatment (→ reluctance to disclose but feeling obligation to disclose).
Paterson et al (2006)[256]	Canada	33 HCV clinic patients	Qualitative study involving individual interviews	Frustration with model of care with dominance in acute rather than chronic care; >50% used CAM to assume control & not need doctors (who they felt delegitimised their knowledge & experience of HCV).
Schackman et al (2008)[257]	US	93 HCV patients & 23 treating health professionals	Quantitative study, involving surveys, demographic & clinical data	Low QoL in HCV; anticipated Tx side effects rated worse by patients than providers; 1-yr of side effects rated equal to 4 yrs of mild HCV symptoms if returned to current health status post Tx, and 2 yrs if return to population norms. 73% willing to risk life on 'certain cure' (standard gamble).
Taylor-Young & Heldebrandt (2009)[258]	US	One person undergoing HCV treatment	Qualitative study involving series of in-depth interviews	Four themes: transition or change; maintaining control of one's life; social support & communication; side effects.
Zickmund et al (2004)[259]	US	322 HCV clinic patients	Mixed methods, involving surveys, demographic & clinical data, & semi-structured interviews	Poor communication with doctors in 40% (re ↓doctor skill, poor doctor HCV management; feelings of misdiagnosis, misleading advice or abandoned, stigmatisation by doctor). Patient psychosocial problems predicted by communication difficulties.
<u>Treatment decisions:</u>				
Khokhar & Lewis (2007)[260]	US	280 patients declining treatment	Quantitative study involving interrogation of a clinical database	Reasons for declining Tx: 44% asymptomatic; 22% side effect concerns; 20% comorbid conditions; 10% ↓social/financial support; 4% efficacy doubts. 79% confirmed ongoing satisfaction with decision 12 months later.
McNally et al (2006)[261]	Australia	224 HCV^+ people	Quantitative study involving surveys on Tx & diagnosis experience, health service access & demographic data	46% reported health 'fair to poor' & HCV symptoms in 63% (only 47% seeing GP); side effects most important in declining & adhering toTx; efficacy most important in deciding to try Tx; diagnosed <5 years most likely to try Tx.
Ogawa and Bova (2009)[262]	US	31 HCV-HIV co-infected	Qualitative study invol-	HCV Tx not a priority; a topic

Authors (year)	Country	Participants	Methodological approach	Outcomes and conclusions
		IDU	ving semi-structured interviews	of fear (might reintroduce injecting) and misinformation (unaware of role of alcohol); others recommended to other IDU to 'get clean & try it'. Fragile nature of 'recovery' from substance use.
Rowan et al (2007)[263]	US	12 male HCV veterans - Tx after delay due to alcohol or depression.	Qualitative study involving focus groups.	Decision making in 4 categories; health care processes; education re Tx contraindications; communication; health behaviours. Most satisfied with Tx decision making process & made changes →Tx start.
Treloar & Hopwood (2008)[264]	Australia	20 HCV patients on Tx & 6 health care workers	Qualitative study involving semi-structured interviews	'Unrealistic optimism' in both patients & health care workers; patients didn't think pre-Tx info was relevant to them & would not experience side effects → delays in treatment for mental health problems.

* study also included in other study category

Abbreviations: **HCV** – hepatitis C virus; **IDU** – injection drug user; **QoL** – quality of life; **Tx** – treatment;

REFERENCES

1. Backmund M, Meyer K, Edlin BR. Infrequent reinfection after successful treatment for hepatitis C virus infection in injection drug users. *Clin Infect Dis*. 2004;39(10):1540-3.
2. Cox AL, Netski DM, Mosbruger T, Sherman SG, Strathdee S, Ompad DC, et al. Prospective evaluation of community-acquired acute-phase hepatitis C virus infection. *Clin Infect Dis*. 2005;40:951-8.
3. Currie SL, Ryan JC, Tracy D, Wright TL, George S, McQuaid R, et al. A prospective study to examine persistent HCV reinfection in injection drug users who have previously cleared the virus. *Drug Alcohol Depend*. 2008;93:148-54.
4. Foley S, Abou-Saleh MT. Risk behaviors and transmission of Hepatitis C in injecting drug users. *Addict Disord Their Treatment*. 2009;8(1):813-21.
5. Fuller CM, Ompad DC, Galea S, Wu Y, Koblin B, Vlahov D. Hepatitis C incidence - a comparison between injection and noninjection drug users in New York City. *J Urban Health*. 2004;81(1):20-4.
6. Grebely J, Conway B, Raffa JD, Lai C, Krajden M, Tyndall MW. Hepatitis C virus reinfection in injection drug users. *Hepatology*. 2006;44(5):1139-45.
7. Hahn JA, Page-Shafer K, Lum PJ, Bourgois P, Stein E, Evans JL, et al. Hepatitis C virus seroconversion among young injection drug users: relationships and risks. *J Infect Dis*. 2002;186:1558-64.
8. Hallinan R, Byrne A, Amin J, Dore GJ. Hepatitis C virus incidence among injecting drug users on opioid replacement therapy. *Aust N Z J Public Health*. 2004;28:576-8.
9. Holtzman D, Barry V, Ouellet LJ, Jarlais DCD, Vlahov D, Golub ET, et al. The influence of needle exchange programs on injection risk behaviors and infection with hepatitis C virus among young injection drug users in select cities in the United States, 1994-2004. *Prev Med*. 2009 Jul;49(1):68-73.
10. Jittiwutikarn J, Thongsawat S, Suriyanon V, Maneekarn N, Celentano D, Razak M, et al. Hepatitis C Infection among drug users in Northern Thailand. *Am J Trop Med Hyg*. 2006;74(6):1111-6.
11. Judd A, Hickman M, Jones S, McDonald T, Parry JV, Stimson GV, et al. Incidence of hepatitis C virus and HIV among new injecting drug users in London: prospective cohort study. *Br Med J*. 2005 Jan 1;330(7481):24-5.
12. Kim C, Kerr T, Li K, Zhang R, Tyndall MW, Montaner JSG, et al. Unstable housing and hepatitis C incidence among injection drug users in a Canadian setting - art. no. 270. *BMC Public Health*. 2009 Jul;9:270-.
13. Maher L, Li J, Jalaludin B, Chant KG, Kaldor JM. High hepatitis C incidence in new injecting drug users: a policy failure? *Aust N Z J Public Health*. 2007;31(1):30-5.
14. Micallef JM, Macdonald V, Jauncey M, Amin J, Rawlinson W, van Beek I, et al. High incidence of hepatitis C virus reinfection within a cohort of injecting drug users. *J Viral Hepat*. 2007 Jun;14(6):413-8.
15. Page K, Hahn JA, Evans J, Shiboski S, Lum P, Delwart E, et al. Acute hepatitis C virus infection in young adult injection drug users: a prospective study of incident infection, resolution, and reinfection. *J Infect Dis*. 2009;200:1216-26.
16. Roy E, Boudreau J-F, Boivin J-F. Hepatitis C virus incidence among young street-involved IDUs in relation to injection experience. *Drug Alcohol Depend*. 2009 Jun 1;102(1-3):158-61.
17. Ruan Y, Qin G, Yin L, Chen K, Qian H-Z, Hao C, et al. Incidence of HIV, hepatitis C and hepatitis B viruses among injection drug users in southwestern China: a 3-year follow-up study. *AIDS*. 2007 Dec;21 Suppl 8:S39-46.
18. van den Berg C, van de Laar TJW, Kok A, Zuure FR, Coutinho RA, Prins M. Never injected, but hepatitis C virus-infected: a study among self-declared never-injecting drug users from the Amsterdam Cohort Studies. *J Viral Hepat*. 2009 Aug;16(8):568-77.

19. González R, Soza A, Hernández V, Pérez RM, Alvarez M, Morales A, et al. Incidence and prevalence of hepatitis C virus infection in Chile. *Ann Hepatol*. 2005;4(2):127-30.
20. Grebely J, Raffa JD, Lai C, Krajden M, Kerr T, Fischer B, et al. Low uptake of treatment for hepatitis C virus infection in a large community-based study of inner city residents. *J Viral hepat*. 2009 May;16(5):352-8.
21. Kondili LA, Chionne P, Costantino A, Villano U, Lo Noce C, Pannozzo F, et al. Infection rate and spontaneous seroreversion of anti-hepatitis C virus during the natural course of hepatitis C virus infection in the general population. *Gut*. 2002;50:693-6.
22. Mazzeo C, Azzaroli F, Giovanell S, Dormi A, Festi D, Colecchia A, et al. Ten year incidence of HCV infection in northern Italy and frequency of spontaneous viral clearance. *Gut*. 2003;52:1030-4.
23. Okayama A, Stuver SO, Tabor E, Tachibana N, Kohara M, Mueller NE, et al. Incident hepatitis C virus infection in a community-based population in Japan. *J Viral Hepatol*. 2002;9(1):43-51.
24. Di Napoli A, Pezzotti P, Di Lallo D, Petrosillo N, Trivelloni C, Di Giulio S, et al. Epidemiology of hepatitis C virus among long-term dialysis patients: a 9-year study in an Italian region. *Am J Kidney Dis*. 2006;48(4):629-37.
25. Furusyo N, Hayashi J, Kakuda K, Ariyama I, Kanmoto-Tanaka Y, Shimizu C, et al. Acute hepatitis C among Japanese hemodialysis patients: a prospective 9-year study. *Am J Gastroenterol*. 2001;96(5):1592-600.
26. Kumagai J, Komiya Y, Tanaka J, Katayama K, Tatsukawa Y, Yorioka N, et al. Hepatitis C virus infection in 2,744 hemodialysis patients followed regularly at nine centers in Hiroshima during November 1999 through February 2003. *J Med Virol*. 2005;76:498-502.
27. Moreira R, Pinho JRR, Fares J, Oba IT, Cardoso MR, Saraceni CP, et al. Prospective study of hepatitis C virus infection in hemodialysis patients by monthly analysis of HCV RNA and antibodies. *Can J Microbiol*. 2003;49:503-7.
28. Ross RS, Viazov S, Clauberg R, Wolters B, Fengler I, Eveld K, et al. Lack of *de novo* hepatitis C virus infections and absence of nosocomial transmissions of GB virus C in a large cohort of German haemodialysis patients. *J Viral Hepat*. 2009;16:230-8.
29. Mikhail NN, Lewis DL, Omar N, Taha H, El-Badawy A, Abdel-Mawgoud N, et al. Prospective study of cross-infection from upper-GI endoscopy in a hepatitis C-prevalent population. *Gastrointest Endosc*. 2007 Apr;65(4):584-8.
30. Mast EE, Hwang L-Y, Seto DSY, Nolte FS, Nainan OV, Wurtzel H, et al. Risk factors for perinatal transmission of hepatitis C virus (HCV) and the natural history of HCV infection acquired in infancy. *J Infect Dis*. 2005 Dec 1;192(11):1880-9.
31. Mok J, Pembrey L, Tovo PA, Newell ML, European Paediatric Hepatitis CVN. When does mother to child transmission of hepatitis C virus occur? *Arch Dis Child Fetal Neonatal Ed*. 2005 Mar;90(2):F156-60.
32. Saleh DA, Shebl F, Abdel-Hamid M, Narooz S, Mikhail N, El-Batonony M, et al. Incidence and risk factors for hepatitis C infection in a cohort of women in rural Egypt. *Trans R Soc Trop Med Hyg*. 2008;102:921-8.
33. Sbebl FM, El-Kamary SS, Saleh DaA, Abdel-Hamid M, Mikhail N, Allam A, et al. Prospective cohort study of mother-to-infant infection and clearance of hepatitis C in rural Egyptian villages. *J Med Virol*. 2009 Jun;81(6):1024-31.
34. Augenbraun M, Goedert JJ, Thomas D, Feldman J, Seaberg EC, French AL, et al. Incident hepatitis C virus in women with human immunodeficiency virus infection. *Clin Infect Dis*. 2003;37:1357-64.
35. Palacios R, Mata R, Aguilar I, Muñoz L, José Ríos M, Vergar S, et al. High seroprevalence but low incidence of HCV infection in a cohort of patients with sexually transmitted HIV in Andalusia, Spain. *J Int Assoc Physicians AIDS Care*. 2009;8(2):100-5.
36. Rauch A, Rickenbach M, Weber R, Hirschel B, Tarr PE, Bucher HC, et al. Unsafe sex and

increased incidence of hepatitis C virus infection among HIV-infected men who have sex with men: the Swiss HIV Cohort Study. *Clin Infect Dis*. 2005;41(3):395-402.
37. Alary M, Joly JR, Vincelette J, Lavoie R, Turmel B, Remis RS. Lack of evidence of sexual transmission of hepatitis C virus in a prospective cohort study of men who have sex with men. *Am J Public Health*. 2005 Mar;95(3):502-5.
38. Ruan YH, Jia YJ, Zhang XX, Liang HY, Li QC, Yang Y, et al. Incidence of HIV-1, Syphilis, Hepatitis B, and Hepatitis C Virus Infections and Predictors Associated With Retention in a 12-Month Follow-Up Study Among Men Who Have Sex With Men in Beijing, China. *J Acquir Immune Defic Syndr*. 2009 Dec;52(5):604-10.
39. Champion JK, Taylor A, Hutchinson S, Cameron S, McMenamin J, Mitchell A, et al. Incidence of hepatitis C virus infection and associated risk factors among Scottish prison inmates: a cohort study. *Am J Epidemiol*. 2004;159(5):514-9.
40. Christensen PB, Krarup HB, Niesters HGM, Norder H, Georgsen J. Prevalence and incidence of bloodborne viral infections among Danish prisoners. *Eur J Epidemio*. 2000;16:1043-9.
41. Dolan KA, Shearer J, White B, Zhou J, Kaldor J, Wodak AD. Four-year follow-up of imprisoned male heroin users and methadone treatment: mortality, re-incarceration and hepatitis C infection. *Addiction*. 2005;100:820-8.
42. Miller ER, Bi P, Ryan P. HCV infection in South Australian prisoners: seroprevalence, seroconversion and risk factors. *Int J Infect Dis*. 2009;13:201-8.
43. Bruno S, Crosignani A, Maisonneuve P, Rossi S, Silini E, Mondelli MU. Hepatitis C virus genotype 1b as a major risk factor associated with hepatocellular carcinoma in patients with cirrhosis: a seventeen-year prospective cohort study.[see comment]. *Hepatology*. 2007 Nov;46(5):1350-6.
44. Bruno S, Zuin M, Crosignani A, Rossi S, Zadra F, Roffi L, et al. Predicting Mortality Risk in Patients With Compensated HCV-Induced Cirrhosis: A Long-Term Prospective Study. *Am J Gastroenterol*. 2009 May;104(5):1147-58.
45. Casiraghi MA, De Paschale M, Romanò L, Biffi R, Assi a, Binelli G, et al. Long-term outcome (35 years) of hepatitis C after acquisition of infection through mini transfusions of blood given at birth. *Hepatology*. 2004;39:90-6.
46. Delladetsima I, Psichogiou M, Sypsa V, Psimenou E, Kostakis A, Hatzakis A, et al. The course of hepatitis C virus infection in pretransplantation anti-hepatitis C virus-negative renal transplant recipients: a retrospective follow-up study. *Am J Kidney Dis*. 2006;47(2):309-16.
47. Federici AB, Santagostino E, Rumi MG, Russo A, Mancuso ME, Soffredini R, et al. The natural history of hepatitis C virus infection in Italian patients with von Willebrand's disease: a cohort study. *Haematologica*. 2006 Apr;91(4):503-8.
48. Fong TL, Hou L, Hutchinson IV, Cicciarelli JC, Cho YW. Impact of hepatitis C infection on outcomes after heart transplantation. *Transplantation*. 2009;88(9):1137-41.
49. Forns X, Amprudanès S, Sanchez-Tapias JM, Guilera M, Sans M, Sánchez-Fueyo A, et al. Long-term follow-up of chronic hepatitis C in patients diagnosed at a tertiary-care center. *J Hepatol*. 2001;35:265-71.
50. Harris HE, Ramsay ME, Andrews NJ, on behalf of the HCV National Register Steering Group. Survival of a national cohort of hepatitis C virus infected patients, 16 years after exposure. *Epidemiol Infect*. 2006;134:472-7.
51. Hirofumi U, Sherri OS, Katsuhiro H, Kotaro K, Fumisato S, Shuji K, et al. Increased rate of death related to presence of viremia among hepatitis C virus antibody-positive subjects in a community-based cohort study. 2009;50(2):393-9.
52. Hui C-K, Zhang H-Y, Shek T, Yao H, Yeung Y-H, Leung K-W, et al. Disease progression in Chinese chronic hepatitis C patients with persistently normal alanine aminotransferase levels. *Aliment Pharmacol Ther*. 2007;25:1283-92.
53. Ikeda K, Arase Y, Kawamura Y, Yatsuji H, Sezaki H, Hosaka T, et al. Necessities of interferon therapy in elderly patients with chronic hepatitis C. *Am J Med*. 2009;122:479-86.

54. Jauncey M, Micallef JM, Gilmour S, Amin J, White PA, Rawlinson W, et al. Clearance of hepatitis C after newly acquired infection in injection drug users. *J Infect Dis*. 2004;190:1270-4.
55. Keating S, Coughlan S, Connell J, Sweeny B, Keenan E. Hepatitis C viral clearance in an intravenous drug-using cohort in the Dublin area. *Ir J Med Sci*. 2005;174(1):37-41.
56. Kumada T, Toyoda H, Kiriyama S, Sone Y, Tanikawa M, Hisanaga Y, et al. Long-Term Follow-Up of Patients With Hepatitis C With a Normal Alanine Aminotransferase. *J Med Virol*. 2009 Mar;81(3):446-51.
57. Martinot-Peignoux M, Boyer N, Cazals-Hatem D, Pham B-N, Gervais A, Le Breton V, et al. Prospective study on anti-hepatitis C virus-positive patients with persistently normal serum alanine transaminase with or without detectable serum hepatitis C virus RNA. *Hepatology*. 2001;34:1000-5.
58. Miyakama K, Tarao K, Ohshige K, Morinaga S, Ohkawa S, Okamoto N, et al. High serum alanine aminotransferase levels for the first three succcessive years can predict very high incidence of hepatocellular carcinoma in patients with Child Stage A HCV-associated liver cirrhosis. *Scand J Gastroenterol*. 2009;44:1340-8.
59. Monica F, Lirussi F, Pregun I, Vasile f, Fabris L, Okolicsanyi L. Hepatitis C virus infection in a resident elderly population: a 10-year follow-up study. *Dig Liver Dis*. 2006;38:336-40.
60. Nagao Y, Tanaka K, Kobayashi K, Kumashiro R, Sata M. A cohort study of chronic liver disease in an HCV hyperendemic area of Japan: a prospective analysis for 12 years. *Int J Mol Med*. 2004;13(2):257-65.
61. Nakagawa H, Maeda S, Yashida H, Tateishi R, Masuzaki R, Ohki T, et al. Serum IL-6 levels and the risk for hepatocarcinogenesis in chronic hepatitis C patients: an analysis based on gender differences. *Int J Cancer*. 2009;125:2264-9.
62. Neal KR, on behalf of the Trent Hepatitis C Study Group. Excess mortality rates in a cohort of patients infected with the hepatitis C virus: a prospective study.[see comment]. *Gut*. 2007 Aug;56(8):1098-104.
63. Okuda K, Yokosuka O. Natural history of chronic hepatitis C in patients on hemodialysis: case control study with 4-23 years of follow-up. *World J Gastroenterol*. 2004;10(15):2209-12.
64. Pergam SA, Wang CC, Gardella CM, Sandison TG, Phipps WT, Hawes SE. Pregnancy complications associated with hepatitis C: data from a 2003-2005 Washington state birth cohort. *Am J Obstet Gynecol*. 2008 Jul;199(1):38.e1-9.
65. Persico M, Perrotta S, Persico E, Terracciano L, Folgori a, Ruggeri L, et al. Hepatitis C virus carriers with persistently normal ALT levels: biological peculiarities and update of the natural history of liver disease at 10 years. *J Viral Hepat*. 2006;13:290-196.
66. Posthouwer D, Makris M, Yee TT, Fischer K, van Veen JJ, Griffioen A, et al. Progression to end-stage liver disease in patients with inherited bleeding disorders and hepatitis C: an international, multicenter cohort study. *Blood*. 2007 May 1;109(9):3667-71.
67. Pradat P, Tillmann HL, Sauleda S, Braconier J-H, Saracco G, Thursz M, et al. Long-term follow-up of the hepatitis C HENCORE cohort: response to therapy and occurrence of liver-related complications. *J Viral Hepat*. 2007;14:556-63.
68. Prasad L, Spicher VM, Negro F, Rickenbach M, Zwahlen M. Little evidence that hepatitis C virus leads to a higher risk of mortality in the absence of cirrhosis and excess alcohol intake: the Swiss Hepatitis C Cohort Study. *J Viral Hepat*. 2009 Sep;16(9):644-9.
69. Punyagupta S, Siwadune T. The long term outcome of thirty eight post-transfusion hepatitis C. *J Med Assoc Thai*. 2001;84(1):12-8.
70. Rumi MG, De Filippi F, La Vecchia C, Donato MF, Gallus S, Del Ninno E, et al. Hepatitis C reactivation in patients with chronic infection with genotypes 1b and 2c: a retrospective cohort study of 206 untreated patients. *Gut*. 2005 Mar;54(3):402-6.
71. Sangiovanni A, Prati GM, Fasani P, Ronchi G, Romeo R, Manini M, et al. The natural

history of compensated cirrhosis due to hepatitis C virus: A 17-year cohort study of 214 patients. *Hepatology*. 2006 Jun;43(6):1303-10.
72. Sanyal AJ, Banas C, Sargeant C, Luketic VA, Sterling RK, Stravitz RT, et al. Similarities and differences in outcomes of cirrhosis due to nonalcoholic steatohepatitis and hepatitis C. *Hepatology*. 2006;43:982-689.
73. Solá R, Álvarez MA, Ballesté B, Montoliu S, Rivera M, Miquel M, et al. Probability of liver cancer and survival in HCV-related or alcoholic-decompensated cirrhosis. A study of 377 patients. *Liver Int*. 2006;26:62-72.
74. Strauss R, Törner A, Duberg AS, Hultcrantz R, Ekdahl K. Hepatocellular carcinoma and other primary liver cancers in hepatitis C patients in Sweden - a low endemic country. *J Viral Hepat*. 2008;15(7):531-7.
75. Tarao K, Rino Y, Ohkawa S, Endo O, Miyakawa K, Tamai S, et al. Sustained low alanine aminotransferase levels can predict the survival for 10 years without hepatocellular carcinoma development in patients with hepatitis C virus-associated liver cirrhosis of Child Stage A. *Intervirology*. 2004;47:65-71.
76. Toshikuni N, Izumi A, Nishino K, Inada N, Sakanoue R, Yamato R, et al. Comparison of outcomes between patients with alcoholic cirrhosis and those with hepatitis C virus-related cirrhosis. *J Gastroenterol Hepatol*. 2009;24:1276-83.
77. Töz H, Nart D, Turan I, Ersöz G, Seziş M, Aşçi G, et al. The acquisition time of infection: a determinant of the severity of hepatitis C virus-related liver disease in renal transplant patients. *Clin Transplant*. 2009 Sep-Oct;23(5):723-31.
78. Tsui JI, Vittinghoff E, Shlipak MG, Bertenthal D, Inadomi J, Rodriguez RA, et al. Association of hepatitis C seropositivity with increased risk for developing end-stage renal disease. *Arch Intern Med*. 2007 Jun 25;167(12):1271-6.
79. Uto H, Stuver SO, Hayashi K, Kumagai K, Sasaki F, Kanmura S, et al. Increased rate of death related to presence of viremia among hepatitis C virus antibody-positive subjects in a community-based cohort study.[see comment]. *Hepatology*. 2009 Aug;50(2):393-9.
80. Wilson LE, Torbenson M, Astemborski J, Faruki H, Spoler C, Rai R, et al. Progression of liver fibrosis among injection drug users with chronic hepatitis C. *Hepatology*. 2006;43:788-95.
81. Wright M, Goldin R, Fabre a, Lloyd J, Thomas H, Trepo C, et al. Measurement and determinants of the natural history of liver fibrosis in hepatitis C virus infection: a cross sectional and longitudinal study. *Gut*. 2003;52:574-9.
82. Yawn BP, Wollan P, Gazzuola L, Kim RW. Diagnosis and 10-year follow-up of a community-based hepatitis C cohort. *J Fam Prac*. 2002;51(2):135-40.
83. Adachi S, Shibuya A, Miura Y, Takeuchi A, Nakazawa T, Saigenji K. Impact of occult hepatitis B virus infection and prior hepatitis B virus infection on development of hepatocellular carcinoma in patients with liver cirrhosis due to hepatitis C virus. *Scand J Gastroenterol*. 2008;43(7):849-56.
84. Amin J, Dore GJ, O'Connell DL, Bartlett M, Tracey E, Kaldor JM, et al. Cancer incidence in people with hepatitis B or C infection: a large community-based linkage study. *J Hepatol*. 2006;45:197-203.
85. Duberg A-S, Törner A, Daviosdóttir L, Aleman S, Blaxhult A, Svensson Å, et al. Cause of death in individuals with chronic HBV and/or HCV infection, a nationwide community-based register study. *J Viral Hepat*. 2008;15:538-50.
86. Fujiwara A, Sakaguchi K, Fujioka S, Iwasaki Y, Senoh T, Nishimura M, et al. Fibrosis progression rates between chronic hepatitis B and C patients with elevated alanine aminotransferase levels. *J Gastroenterol*. 2008;43(6):484-91.
87. Gannon CJ, Izzo F, Aloia TA, Pignata S, Nasti G, Vallone P, et al. Can hepatocellular cancer screening increase the proportion of long-term survivors? *Hepatogastroenterol*. 2009;56(93):1152-6.

88. Ikeda K, Arase Y, Kobayashi M, Saitoh S, Someya T, Hosaka T, et al. Significance of multicentric cancer recurrence after potentially curative ablation of hepatocellular carcinoma: a longterm cohort study of 892 patients with viral cirrhosis. *J Gastroenterol*. 2003;38:865-76.
89. Ikeda K, Marusawa H, Osaki Y, Nakamura T, Kitajima N, Yamashita Y, et al. Antibody to hepatitis B core antigen and risk for hepatitis C-related hepatocellular carcinoma: a prospective study. *Ann Intern Med*. 2007;146:649-56.
90. Inoue M, Kurahashi N, Iwasaki M, Tanaka Y, Mizokami M, Noda M, et al. Metabolic factors and subsequent risk of hepatocellular carcinoma by hepatitis virus infection status: a large-scale population-based cohort study of Japanese men and women (JPHC Study Cohort II). *Cancer Causes Control*. 2009 Jul;20(5):741-50.
91. Mori M, Hara M, Wada I, Hara T, Yamamoto K, Honda M, et al. Prospective study of hepatitis B and C viral infections, cigarette smoking, alcohol consumption, and other factors associated with hepatocellular carcinoma risk in Japan. *Am J Epidemiol*. 2000;151(2):131-9.
92. Sasaki Y, Yamada T, Tanaka H, Ohigashi H, Eguchi H, Yano M, et al. Risk of recurrence in a long-term follow-up after surgery in 417 patients with hepatitis B-or hepatitis C-related hepatocellular carcinoma. *Ann Surg*. 2006;244:771-80.
93. Sun CA, Wu DM, Lin CC, Lu SN, You SL, Wang LY, et al. Incidence and cofactors of hepatitis C virus-related hepatocellular carcinoma: a prospective study of 12,008 men in Taiwan. *Am J Epidemiol*. 2003;157(8):674-82.
94. Tanaka H, Tsukuma H, Yamano H, Oshima A, Shibata H. Prospective study on the risk of hepatocellular carcinoma among hepatitis C virus-positive blood donors focusing on demographic factors, alanine aminotransferase level at donation and interaction with hepatitis B virus. *Int J Cancer*. 2004;112:1075-80.
95. Tanaka K, Nagao Y, Ide t, Kumashiro R, Sata M. Antibody to hepatitis B core antigen is associated with the development of hepatocellular carcinoma in hepatitis C virus-infected persons: a 12-year prospective study. *Int J Mol Med*. 2006;17:827-32.
96. Wang C-S, Wang S-T, Yao W-J, Chang T-T, Chou P. Hepatitis C virus infection and the development of type 2 diabetes in a community-based longitudinal study. *Am J Epidemiol*. 2007;166(2):196-203.
97. Zampino R, Marrone A, Merola A, Trani B, Cirillo G, Karayiannis P, et al. Long-term outcome of hepatitis B and hepatitis C virus co-infection and single HBV infection acquired in youth. *J Med Virol*. 2009;81:2012-20.
98. Anderson KB, Guest JL, Rimland D. Hepatitis C virus coinfection increases mortality in HIV-infected patients in the highly active antiretroviral therapy era: data from the HIV Atlanta VA Cohort study. *Clin Infect Dis*. 2004;39:1507-13.
99. Cheng DM, Nunes D, Libman H, Vidaver J, Alperen JK, Saitz R, et al. Impact of hepatitis C on HIV progression in adults with alcohol problems. *Alcohol Clin Exp Res*. 2007;31(5):829-36.
100. García-García JA, Romero-Gómez M, Girón-González JA, Rivera-Irigoin R, Torre-Cisneros J, Montero JL, et al. Incidence of and factors associated with hepatocellular carcinoma among hepatitis C virus and Human Immunodeficiency virus coinfected patients with decompensated cirrhosis. *AIDS Res Human Retroviruses*. 2006;22(12):1236-41.
101. Giordano TP, Kramer JR, Souchek J, Richardson P, El-Serag HB. Cirrhosis and hepatocellular carcinoma in HIV-infected veterans with and without the hepatitis C virus: a cohort study, 1992-2001. *Arch Intern Med*. 2004 Nov 22;164(21):2349-54.
102. Grebely J, Raffa JD, Lai C, Krajden M, Conway B, Tyndall MW. Factors associated with spontaneous clearance of hepatitis C virus among illicit drug users. *Can J Gastroenterol*. 2007 Jul;21(7):447-51.
103. Hansen N, Obel N, Christensen PB, Krarup H, Laursen AL, Clausen MR, et al. Predictors of antiviral treatment initiation in hepatitis C virus-infected patients: a Danish cohort study. *J Viral Hepat*. 2009 Sep;16(9):659-65.

104. Lumbreras B, Jarrín I, del Amo J, Pérez-Hoyos S, Muga R, García-d la Hear M, et al. Impact of hepatitis C infection on long-term mortality of injecting drug users from 1990 to 2002: differences before and after HAART. *AIDS*. 2006 Jan 2;20(1):111-6.
105. Merchante N, Giron-Gonzalez JA, Gonzalez-Serrano M, Torre-Cisneros J, Garcia-Garcia JA, Arizcorreta A, et al. Survival and prognostic factors of HIV-infected patients with HCV-related end-stage liver disease. *AIDS*. 2006 Jan 2;20(1):49-57.
106. Pineda JA, Aguilar-Guisado M, Rivero A, Giron-Gonzalez JA, Ruiz-Morales J, Merino D, et al. Natural History of Compensated Hepatitis C Virus-Related Cirrhosis in HIV-Infected Patients. *Clin Infect Dis*. 2009 Oct;49(8):1274-82.
107. Smit C, van den Berg C, Geskus R, Berkhout B, Coutinho R, Prins M. Risk of hepatitis-related mortality increased among hepatitis C virus/HIV-coinfected drug users compared with drug users infected only with hepatitis C virus: a 20-year prospective study. *J Acquir Immune Defic Syndr*. 2008 Feb 1;47(2):221-5.
108. Weis N, Lindhardt BØ, Hansen A-BE, Laursen AL, Christensen PB, Nielsen H, et al. Impact of hepatitis C virus coinfection on response to highly active antiretroviral therapy and outcome in HIV-infected individuals: a nationwide cohort study. *Clin Infect Dis*. 2006;42:1481-7.
109. Bonacini M, Louie S, Bzowej N, Wohl AR. Survival in patients with HIV infection and viral hepatitis B or C: a cohort study. *AIDS*. 2004;18(15):2039-45.
110. Melendez-Morales L, Konkle BA, Preiss L, Zhang M, Mathew P, Eyster ME, et al. Chronic hepatitis B and other correlates of spontaneous clearance of hepatitis C virus among HIV-infected people with hemophilia. *AIDS*. 2007 Jul 31;21(12):1631-6.
111. Bonkovsky HL, Snow KK, Malet PF, Back-Madruga C, Fontana RJ, Sterling RK, et al. Health-related quality of life in patients with chronic hepatitis C and advanced fibrosis. *J Hepatol*. 2007 Mar;46(3):420-31.
112. Dan AA, Martin LM, Crone C, Ong JP, Farmer DW, Wise T, et al. Depression, anemia and health-related quality of life in chronic hepatitis C. *J Hepatol*. 2006 Mar;44(3):491-8.
113. Dan AA, Crone C, Wise TN, Martin LM, Ramsey L, Magee S, et al. Anger experiences among hepatitis C patients: relationship to depressive symptoms and health-related quality of life. *Psychosomatics*. 2007 May-Jun;48(3):223-9.
114. Evon DM, Ramcharran D, Belle SH, Terrault NA, Fontana RJ, Fried MW, et al. Prospective analysis of depression during peginterferon and ribavirin therapy of chronic hepatitis C: result of the Virahep-C Study. *Am J Gastroenterology*. 2009;Advance on line publication, 29 September 2009.
115. Falasca K, Mancino P, Ucciferri C, Dalessandro M, Manzoli L, Pizzigallo E, et al. Quality of life, depression, and cytokine patterns in patients with chronic hepatitis C treated with antiviral therapy. *Clin Invest Med*. 2009;32(3):E212-8.
116. Hollander A, Foster GR, Weiland O. Health-related quality of life before, during and after combination therapy with interferon and ribavirin in unselected Swedish patients with chronic hepatitis C. *Scand J Gastroenterol*. 2006 May;41(5):577-85.
117. McHutchison JG, Ware JE, Bayliss MS, Pianko S, Albrecht JK, Cort S, et al. The effects of interferon alpha-2b in combination with ribavirin on health related quality of life and work productivity. *J Hepatol*. 2001 Jan;34(1):140-7.
118. Şahingöz M, Uğuz F, Erayman I, Kaya N, Aribaş ET. Interferon-alpha treatment in patients with chronic viral hepatitis C: The incidence of major depression and changes in quality of life. *Arch Neuropsychiatr*. 2009;45(4):130-4.
119. Bruggmann P, Falcato L, Dober S, Helbling B, Keiser O, Negro F, et al. Active intravenous drug use during chronic hepatitis C therapy does not reduce sustained virological response rates in adherent patients. *J Viral Hepat*. 2008 Oct;15(10):747-52.
120. Chavalitdhamrong D, Tanwandee T. Long-term outcomes of chronic hepatitis C patients with sustained virological response at 6 months after the end of treatment. *World J Gastroenterol*.

2006 Sep 14;12(34):5532-5.
121. Dalgard O, Bjøro K, Hellum K, Myrvang B, Skaug K, Gutigard G, et al. Treatment of chronic hepatitis C in injecting drug users: 5 years' follow-up. *Eur Addict Res*. 2002;8:45-9.
122. Everhart JE, Lok AS, Kim H-Y, Morgan TR, Lindsay KL, Chung RT, et al. Weight-related effects on disease progression in the Hepatitis C Antiviral Long-Term Treatment against Cirrhosis Trial. *Gastroenterology*. 2009;137:549-57.
123. Goodman ZD, Stoddard AM, Bonkovsky HL, Fontana RJ, Ghany MG, Morgan TR, et al. Fibrosis progression in chronic hepatitis C: morphometric image analysis in the HALT-C Trial. *Hepatology*. 2009;50:1738-49.
124. Ikeda M, Fujiyama S, Tanaka M, Sata M, Ide T, Yatsuhashi H, et al. Risk factors for development of hepatocellular carcinoma in patients with chronic hepatitis C after sustained response to interferon. *J Gastroenterol*. 2005 Feb;40(2):148-56.
125. Imazeki F, Yokosuka O, Fukai K, Saisho H. Favorable prognosis of chronic hepatitis C after interferon therapy by long-term cohort study. *Hepatology*. 2003;38:493-502.
126. Kobayashi S, Takeda T, Enomoto M, Tamori A, Kawada N, Habu D, et al. Development of hepatocellular carcinoma in patients with chronic hepatitis C who had a sustained virological response to interferon therapy: a multicenter, retrospective cohort study of 1124 patients. *Liver Int* 2007 Mar;27(2):186-91.
127. Manesis EK, Papatheodoridis GV, Touloumi G, Karfoulidous A, Ketikoglou J, Kitis GE, et al. Natural course of treated and untreated chronic HCV infection: results of the nationwide Hepnet.Greece cohort study. *Aliment Pharmacol Ther*. 2009;29:1121-30.
128. Okanoue T, Minami M, Makiyama A, Sumida Y, Yassui K, Itoh Y. Natural course of asymptomatic hepatitis C virus-infected patients and hepatocellular carcinoma after interferon therapy. *Clin Gastroenterol Hepatol*. 2005;3 (Suppl 2):S89-S91.
129. Veldt BJ, Heathcote EJ, Wedemeyer H, Reichen J, Hofmann WP, Zeuzem S, et al. Sustained virologic response and clinical outcomes in patients with chronic hepatitis C and advanced fibrosis.[summary for patients in Ann Intern Med. 2007 Nov 20;147(10):I47; PMID: 18025441]. *Arch Intern Med*. 2007 Nov 20;147(10):677-84.
130. De Bona A, Galli L, Gallotta G, Guzzo A, Alagna L, Lazzarin A, et al. Rate of cirrhosis progression reduced in HIV/HCV co-infected non-responders to anti-HCV therapy. *New Microbiol*. 2007;30(3):259-64.
131. Scott JD, Wald A, Kitahata M, Krantz E, Drolette L, Corey L, et al. Hepatitis C Virus Is Infrequently Evaluated and Treated in an Urban HIV Clinic Population. *AIDS Patient Care STDS*. 2009 Nov;23(11):925-9.
132. Zinkernagel AS, von Wyl V, Ledergerber B, Rickenbach M, Furrer H, Battegay M, et al. Eligibility for and outcome of hepatitis C treatment of HIV-coinfected individuals in clinical practice: the Swiss HIV cohort study. *Antivir Ther*. 2006;11(2):131-42.
133. Ananthakrishnan AN, Saeian K. Racial differences in liver transplantation outcomes in the MELD era.[see comment]. *Am J Gastroenterol*. 2008 Apr;103(4):901-10.
134. Bizollon T, Pradat P, Mabrut JY, Chevallier M, Adham M, Radenne S, et al. Benefit of sustained virological response to combination therapy on graft survival of liver transplanted patients with recurrent chronic hepatitis C. *Am J Transplant*. 2005 Aug;5(8):1909-13.
135. Feurer ID, Wright JK, Payne JL, Kain AC, Wise PE, Hale P, et al. Effects of hepatitis C virus infection and its recurrence after liver transplantation on functional performance and health-related quality of life. *J Gastrointest Surg*. 2002 Jan-Feb;6(1):108-15.
136. Gallegos-Orozco JF, Yosephy A, Noble B, Aqel BA, Byrne TJ, Williams JW, et al. Natural history of post-liver transplantation hepatitis C: a review of factors that may influence its course. *Liver Transpl*. 2009;15:1872-81.
137. Hong Z, Smart G, Dawood M, Kaita K, Wen SW, Gomes J, et al. Hepatitis C infection and survivals of liver transplant patients in Canada, 1997-2003. *Transplant Proceed*. 2008 Jun;40(5):1466-70.

138. Selzner M, Kashfi A, Selzner N, McCluskey S, Greig PD, Cattral MS, et al. Recipient age affects long-term outcome and hepatitis C recurrence in old donor livers following transplantation. *Liver Transpl*. 2009;15:1288-95.
139. Takada Y, Haga H, Ito T, Nabeshima M, Ogawa M, Kasahara M, et al. Clinical outcomes of living donor liver transplantation for hepatitis C virus (HCV)-positive patients. *Transplantation*. 2006;81(3):350-4.
140. Verna EC, De Martin E, Burra P, Neri D, Gaglio PJ, Emond JC, et al. The impact of hepatitis C and biliary complications on patient and graft survival following liver transplantation. *Am J Transpl*. 2009;9:1398-405.
141. Bailey DE, Jr., Landerman L, Barroso J, Bixby P, Mishel MH, Muir AJ, et al. Uncertainty, symptoms, and quality of life in persons with chronic hepatitis C. *Psychosomatics*. 2009 Mar-Apr;50(2):138-46.
142. Balfour L, Cooper C, Kowal J, Tasca GA, Silverman A, Kane M, et al. Depression and cigarette smoking independently relate to reduced health-related quality of life among Canadians living with hepatitis C. *Can J Gastroenterol*. 2006 Feb;20(2):81-6.
143. Batista-Neves S, Quarantini LC, Galvao-de Almeida A, Cardeal M, Lacerda AL, Parana R, et al. Impact of psychiatric disorders on the quality of life of brazilian HCV-infected patients. *Braz J Infect Dis*. 2009 Feb;13(1):40-3.
144. Coughlan B, Sheehan J, Hickey A, Crowe J. Psychological well-being and quality of life in women with an iatrogenic hepatitis C virus infection. *Br J Health Psychol*. 2002 Feb;7:105-16.
145. Danoff A, Khan O, Wan DW, Hurst L, Cohen D, Tenner CT, et al. Sexual dysfunction is highly prevalent among men with chronic hepatitis C virus infection and negatively impacts health-related quality of life. *Am J Gastroenterol*. 2006 Jun;101(6):1235-43.
146. Gallegos-Orozco JF, Fuentes AP, Gerardo Argueta J, Perez-Pruna C, Hinojosa-Becerril C, Sixtos-Alonso MS, et al. Health-related quality of life and depression in patients with chronic hepatitis C. *Arch Med Res*. 2003 Mar-Apr;34(2):124-9.
147. Gifford SM, O'Brien ML, Bammer G, Banwell C, Stoové M. Australian women's experiences of living with hepatitis C virus: results from a cross-sectional survey. *J Gastroenterol Hepatol*. 2003;18:841-50.
148. Gifford SM, O'Brien ML, Smith A, Temple-Smith M, Stoové M, Mitchell D, et al. Australian men's experiences of living with hepatitis C virus: results from a cross-sectional survey. *J Gastroenterol Hepatol*. 2005 Jan;20(1):79-86.
149. Gunasekera S, Fraser J, Alexander C. Quality of life in Hepatitis C virus infection: assessment of rural patients living in north-western New South Wales. *Aust J Rural Health*. 2008 Jul;16(4):213-20.
150. Häuser W, Zimmer C, Schiedermaier P, Grandt D. Biopsychosocial predictors of health-related quality of life in patients with chronic hepatitis C. *Psychosom Med*. 2004 Nov-Dec;66(6):954-8.
151. Helbling B, Overbeck K, Gonvers JJ, Malinverni R, Dufour JF, Borovicka J, et al. Host- rather than virus-related factors reduce health-related quality of life in hepatitis C virus infection. *Gut*. 2008 Nov;57(11):1597-603.
152. Hickey AM, McGee HM, Smith M, Murray F. Iatrogenesis and hepatitis C infection: Implications for well-being. *Ir J Psychol*. 2008;29(1-2):19-33.
153. Hussain KB, Fontana RJ, Moyer CA, Su GL, Sneed-Pee N, Lok AS. Comorbid illness is an important determinant of health-related quality of life in patients with chronic hepatitis C. 2001 Sep;96(9):2737-44.
154. Kallman J, O'Neil MM, Larive B, Boparai N, Calabrese L, Younossi ZM. Fatigue and health-related quality of life (HRQL) in chronic hepatitis C virus infection. *Dig Dis Sci*. 2007 Oct;52(10):2531-9.
155. Kang SC, Hwang SJ, Lee SH, Chang FY, Lee SD. Health-related quality of life and impact of

antiviral treatment in Chinese patients with chronic hepatitis C in Taiwan. *World J Gastroenterol*. 2005 Dec 21;11(47):7494-8.
156. Kramer L, Bauer E, Funk G, Hofer H, Jessner W, Steindl-Munda P, et al. Subclinical impairment of brain function in chronic hepatitis C infection. *J Hepatol*. 2002 Sep;37(3):349-54.
157. Kramer L, Hofer H, Bauer E, Funk G, Formann E, Steindl-Munda P, et al. Relative impact of fatigue and subclinical cognitive brain dysfunction on health-related quality of life in chronic hepatitis C infection. *Aids*. 2005 Oct;19:S85-S92.
158. Kwan JW, Cronkite RC, Yiu A, Goldstein MK, Kazis L, Cheung RC. The impact of chronic hepatitis C and co-morbid illnesses on health-related quality of life. *Qual Life Res*. 2008 Jun;17(5):715-24.
159. Lim JK, Cronkite R, Goldstein MK, Cheung RC. The impact of chronic hepatitis C and comorbid psychiatric illnesses on health-related quality of life. *J Clin Gastroenterol*. 2006 Jul;40(6):528-34.
160. Mikoka-Walus AA. Psychological problems in gastroenterology outpatients: a South Australian experience. Psychological co-morbidity in IBD, IBS and hepatitis C. *Clin Pract Epidemiol Ment Health*. 2008;4:15.
161. Miller ER, Hiller JE, Shaw DR. Quality of life in HCV infection: lack of association with ALT levels. *Aust N Z J Public Health*. 2001;25:355-61.
162. Moyer CA, Fontana RJ, Hussain K, Lok ASF, Schwartz S. The role of optimism/pessimism in HRQOL in chronic hepatitis C patients. *J Clin Psychol Med Settings*. 2003 Mar;10(1):41-9.
163. Pojoga C, Dumitrascu DL, Pascu O, Grigorescu M, Radu C, Damian D. Impaired health-related quality of life in Romanian patients with chronic viral hepatitis before antiviral therapy. *Eur J Gastroenterol Hepatol*. 2004 Jan;16(1):27-31.
164. Rowan PJ, Al-Jurdi R, Tavakoli-Tabasi S, Kunik ME, Satrom SL, El-Serag HB. Physical and psychosocial contributors to quality of life in veterans with hepatitis C not on antiviral therapy. *J Clin Gastroenterol*. 2005 Sep;39(8):731-6.
165. Schwarzinger M, Dewedar S, Rekacewicz C, Abd Elaziz KM, Fontanet A, Carrat F, et al. Chronic hepatitis C virus infection: Does it really impact health-related quality of life? A study in rural Egypt. *Hepatology*. 2004 Dec;40(6):1434-41.
166. Teixeira MCD, de Sá Ribeiro MdG, da Costa Gayotto LC, de Alencar Fischer Chamone D, Strauss E. Worse quality of life in volunteer blood donors with hepatitis C. *Transfusion*. 2006 Feb;46(2):278-83.
167. Teuber G, Schafer A, Rimpel J, Paul K, Keicher C, Scheurlen M, et al. Deterioration of health-related quality of life and fatigue in patients with chronic hepatitis C: Association with demographic factors, inflammatory activity, and degree of fibrosis. *J Hepatol*. 2008 Dec;49(6):923-9.
168. von Wagner M, Lee JH, Kronenberger B, Friedl R, Sarrazin C, Teuber G, et al. Impaired health-related quality of life in patients with chronic hepatitis C and persistently normal aminotransferase levels. *J Viral Hepat*. 2006 Dec;13(12):828-34.
169. Dalgard O, Egeland A, Skaug K, Vilimas K, Steen T. Health-related quality of life in active injecting drug users with and without chronic hepatitis C virus infection. *Hepatology*. 2004 Jan;39(1):74-80.
170. Gjeruldsen S, Loge JH, Myrvang B, Opjordsmoen S. Drug addiction in hepatitis C patients leads to a lower quality of life. *Nord J Psychiatry*. 2006;60(2):157-61.
171. Baum MK, Jayaweera DT, Duan R, Sales S, Lai S, Rafie C, et al. Quality of life, symptomatology and healthcare utilization in HIV/HCV co-infected drug users in Miami. *J Addict Dis*. 2008;27(2):37-48.
172. Braitstein P, Montessori V, Chan K, Montaner JS, Schechter MT, O'Shaughnessy MV, et al. Quality of life, depression and fatigue among persons co-infected with HIV and hepatitis C:

outcomes from a population-based cohort. *AIDS Care*. 2005 May;17(4):505-15.
173. Fleming CA, Christiansen D, Nunes D, Heeren T, Thornton D, Horsburgh CR, Jr., et al. Health-related quality of life of patients with HIV disease: impact of hepatitis C coinfection. *Clin Infect Dis*. 2004 Feb 15;38(4):572-8.
174. Ozkan M, Corapcioglu A, Balcioglu I, Ertekin E, Khan S, Ozdemir S, et al. Psychiatric morbidity and its effect on the quality of life of patients with chronic hepatitis B and hepatitis C. *Int J Psychiatr Med*. 2006;36(3):283-97.
175. Svirtlih N, Pavic S, Terzic D, Delic D, Simonovic J, Gvozdenovic E, et al. Reduced quality of life in patients with chronic viral liver disease as assessed by SF12 questionnaire. *J Gastrointestin Liver Dis*. 2008 Dec;17(4):405-9.
176. Thein HH, Maruff P, Krahn M, Kaldor JM, Koorey DJ, Brew BJ, et al. Cognitive function, mood and health-related quality of life in hepatitis C virus (HCV)-monoinfected and HIV/HCV-coinfected individuals commencing HCV treatment. *HIV Med*. 2007;8:192-202.
177. Tsui JI, Bangsberg DR, Ragland K, Hall CS, Riley ED. The impact of chronic hepatitis C on health-related quality of life in homeless and marginally housed individuals with HIV. *AIDS Behav*. 2007;11:603-10.
178. Björnsson E, Verbaan H, Oksanen A, Frydén A, Johansson J, Friberg S, et al. Health-related quality of life in patients with different stages of liver disease induced by hepatitis C. *Scand J Gastroenterol*. 2009;44(7):878-87.
179. Chong C, Gulamhussein A, Heathcote EJ, Lilly L, Sherman M, Naglie G, et al. Health-state utilities and quality of life in hepatitis C patients. *Am J Gastroenterol*. 2003 Mar;98(3):630-8.
180. Córdoba J, Flavià M, Jacas C, Sauleda S, Esteban JI, Vargas V, et al. Quality of life and cognitive function in hepatitis C at different stages of liver disease. *J Hepatol*. 2003 Aug;39(2):231-8.
181. Hsu PC, Krajden M, Yoshida EM, Anderson FH, Tomlinson GA, Krahn MD. Does cirrhosis affect quality of life in hepatitis C virus-infected patients? *Liver Int*. 2009 Mar;29(3):449-58.
182. Afsar B, Elsurer R, Sezer S, Ozdemir NF. Quality of life in hemodialysis patients: hepatitis C virus infection makes sense. *Int Urol Nephrol*. 2009a Dec;41(4):1011-9.
183. Afsar B, Ozdemir NF, Sezer S, Haberal M. Quality of life is not related with liver disease severity but with anemia, malnutrition, and depression in HCV-infected hemodialysis patients. *Hemodial Int*. 2009b Jan;13(1):62-71.
184. Posthouwer D, Plug I, van der Bom JG, Fischer K, Rosendaal FR, Mauser-Bunschoten EP. Hepatitis C and health-related quality of life among patients with hemophilia. *Haematologica*. 2005 Jun;90(6):846-50.
185. Marcellin F, Preau M, Ravaux I, Dellamonica P, Spire B, Carrieri MP. Self-reported fatigue and depressive symptoms as main indicators of the quality of life (QOL) of patients living with HIV and hepatitis C: Implications for clinical management and future research. *HIV Clin Trials*. 2007 Sep-Oct;8(5):320-7.
186. Batki SL, Canfield KM, Smyth E, Ploutz-Snyder R. Health-related quality of life in methadone maintenance patients with untreated hepatitis C virus infection. *Drug Alcohol Depend*. 2009 May 1;101(3):176-82.
187. Bianchi G, Loguercio C, Sgarbi D, Abbiati R, Chen CH, Disalvo D, et al. Reduced Quality of Life in patients with chronic hepatitis C: effects of interferon treatment. *Dig Liver Dis*. 2000 Jun-Jul;32(5):398-405.
188. Chang SC, Ko WS, Wu SS, Peng CY, Yang SS. Factors associated with quality of life in chronic hepatitis C patients who received interferon plus ribavirin therapy. *J Formos Med Assoc*. 2008 Jun;107(6):454-62.
189. Fontana RJ, Hussain KB, Schwartz SM, Moyer CA, Su GL, Lok ASF. Emotional distress in chronic hepatitis C patients not receiving antiviral therapy. *J Hepatol*. 2002 Mar;36(3):401-7.
190. Hilsabeck RC, Hassanein TI, Ziegler EA, Carlson MD, Perry W. Effect of interferon-alpha on cognitive functioning in patients with chronic hepatitis C. *J Int Neuropsychol Soc*. 2005

Jan;11(1):16-22.
191. Hopwood M, Treloar C. The experience of interferon-based treatments for hepatitis C infection. *Qual Health Res*. 2005 May;15(5):635-46.
192. Schäfer A, Wittchen HU, Backmund M, Soyka M, Golz J, Siegert J, et al. Psychopathological changes and quality of life in hepatitis C virus-infected, opioid-dependent patients during maintenance therapy. *Addiction*. 2009 Apr;104(4):630-40.
193. Akyüz F, Beşişik F, Pinarbaşi B, Demir K, Kaymakoğlu ST, Çakaloğlu Y, et al. The quality of life in hemodialysis patients with chronic hepatitis C virus infection. *Turk J Gastroenterol*. 2009 Dec;20(4):243-6.
194. Fontana RJ, Moyer CA, Sonnad S, Lok ASF, Sneed-Pee N, Walsh J, et al. Comorbidities and quality of life in patients with interferon-refractory chronic hepatitis C. *Am J Gastroenterol*. 2001 Jan;96(1):170-8.
195. John-Baptiste AA, Tomlinson G, Hsu PC, Krajden M, Heathcote EJ, Laporte A, et al. Sustained Responders Have Better Quality of Life and Productivity Compared With Treatment Failures Long After Antiviral Therapy for Hepatitis C. *Am J Gastroenterol*. 2009 Oct;104(10):2439-48.
196. Taliani G, Rucci P, Biliotti E, Cirrincione L, Aghemo A, Alberti A, et al. Therapy expectations and physical comorbidity affect quality of life in chronic hepatitis C virus infection. *J Viral Hepat*. 2007;14:875-82.
197. Dudley T, Chaplin D, Clifford C, Mutimer DJ. Quality of life after liver transplantation for hepatitis C infection. *Qual Life Res*. 2007 Oct;16(8):1299-308.
198. Paterson DL, Gayowski T, Wannstedt CF, Wagener MM, Marino IR, Vargas H, et al. Quality of life in long-term survivors after liver transplantation: impact of recurrent viral hepatitis C virus hepatitis. *Clin Transplant*. 2000 Feb;14(1):48-54.
199. Blasiole JA, Shinkunas L, Labrecque DR, Arnold RM, Zickmund SL. Mental and physical symptoms associated with lower social support for patients with hepatitis C. *World J Gastroenterol*. 2006 Aug 7;12(29):4665-72.
200. Carrier N, Laplante J, Bruneau J. Exploring the contingent reality of biomedicine: injecting drug users, hepatitis virus and risk. *Health Risk Soc*. 2005;7(2):123-40.
201. Grundy G, Beeching N. Understanding social stigma in women with hepatitis C. *Nurs Stand*. 2004 Oct 6-12;19(4):35-9.
202. Janke EA, McGraw S, Garcia-Tsao G, Fraenkel L. Psychosocial issues in hepatitis C: a qualitative analysis. *Psychosomatics*. 2008;49(6):494-501.
203. Sgorbini M, O'Brien L, Jackson D. Living with hepatitis C and treatment: the personal experiences of patients. *J Clin Nurs*. 2009 Aug;18(16):2282-91.
204. Temple-Smith M, Gifford S, Stoové M. The lived experience of men and women with hepatitis C: implications for support needs and health information. *Aust Health Rev*. 2004;27(2):46-56.
205. Castera L, Constant A, Bernard PH, de Ledinghen V, Couzigou P. Lifestyle changes and beliefs regarding disease severity in patients with chronic hepatitis C. *J Viral Hepatitis*. 2006b Jul;13(7):482-8.
206. Roy E, Nonn É, Haley N, Cox J. Hepatitis C meaning and preventive strategies among street-involved young injection drug users in Montréal. *Int J Drug Policy*. 2007;18:397-405.
207. Scognamiglio P, Galati V, Navarra A, Longo MA, Aloisi MS, Antonini MG, et al. Impact of hepatitis C virus infection on lifestyle. *World J Gastroenterol*. 2007 May;13(19):2722-6.
208. Soykan A, Boztaş H, İdilman R, Özel ET, Tüzün AE, Özden A, et al. Sexual dysfunctions in HCV patients and its correlations with psychological and biological variables. *Int J Impot Res*. 2005 Mar-Apr;17(2):175-9.
209. Wright NMJ, Tompkins CNE, Jones L. Exploring risk perceptions and behaviour of homeless injecting drug users diagnosed with hepatitis C. *Health Soc Care Community*. 2005;13(1):75-83.

210. Zacks S, Beavers K, Theodore D, Dougherty K, Batey B, Shumaker J, et al. Social stigmatization and hepatitis C virus infection. *J Clin Gastroenterol*. 2006 Mar;40(3):220-4.
211. Bova C, Jaffarian C, Himlan P, Mangini L, Ogawa L. The symptom experience of HIV/HCV-Coinfected adults. *J Assoc Nurses Aids Care*. 2008 May-Jun;19(3):170-80.
212. Conrad S, Garrett LE, Cooksley WGE, Dunne MP, MacDonald GA. Living with chronic hepatitis C means 'you just haven't got a normal life any more'. *Chronic Iln*. 2006;2:121-31.
213. Copeland L. The drug user's identity and how it relates to being hepatitis C antibody positive: a qualitative study. *Drugs Educ Pre Policy*. 2004;11(2):129-47.
214. Dwight MM, Kowdley KV, Russo JE, Ciechanowski PS, Larson AM, Katon WJ. Depression, fatigue, and functional disability in patients with chronic hepatitis C. *J Psychosomat Res*. 2000 Nov;49(5):311-7.
215. Forton DM, Thomas HC, Murphy CA, Allsop JM, Foster GR, Main J, et al. Hepatitis C and cognitive impairment in a cohort of patients with mild liver disease. *Hepatology*. 2002 Feb;35(2):433-9.
216. Glacken M, Kernohan G, Coates V. Diagnosed with Hepatitis C: a descriptive exploratory study. *International Journal of Nursing Studies*. 2001 Feb;38(1):107-16.
217. Glacken M, Coates V, Kernohan G, Hegarty J. The experience of fatigue for people living with hepatitis C. *J Clin Nurs*. 2003 Mar;12(2):244-52.
218. Golden J, O'Dwyer AM, Conroy RM. Depression and anxiety in patients with hepatitis C: prevalence, detection rates and risk factors. *Gen Hosp Psych*. 2005 Nov-Dec;27(6):431-8.
219. Goulding C, O'Connell P, Murray FE. Prevalence of fibromyalgia, anxiety and depression in chronic hepatitis C virus infection: relationship to RT-PCR status and mode of acquisition. *Eur J Gastroenterol Hepatol*. 2001 May;13(5):507-11.
220. Grassi L, Satriano J, Serra A, Biancosino B, Zotos S, Sighinolfi L, et al. Emotional stress, psychosocial variables and coping associated with hepatitis C virus and human immunodeficiency virus infections in intravenous drug users. *Psychother Psychosom*. 2002 Nov-Dec;71(6):342-9.
221. Groessl EJ, Weingart KR, Kaplan RM, Clark JA, Gifford AL, Ho SB. Living with hepatitis C: qualitative interviews with hepatitis C-infected veterans. *J Gen Intern Med*. 2008 Dec;23(12):1959-65.
222. Hassoun Z, Willems B, Deslauriers J, Nguyen BN, Huet PM. Assessment of fatigue in patients with chronic hepatitis C using the fatigue impact scale. *Dig Dis Sci*. 2002 Dec;47(12):2674-81.
223. Hilsabeck RC, Perry W, Hassanein TI. Neuropsychological impairment in patients with chronic hepatitis C. *Hepatology*. 2002 Feb;35(2):440-6.
224. Hilsabeck RC, Hassanein TI, Carlson MD, Ziegler EA, Perry W. Cognitive functioning and psychiatric symptomatology in patients with chronic hepatitis C. *J Int Neuropsychol Soc*. 2003 Sep;9(6):847-54.
225. Hogg RS, Craib KJP, Pi D, Lee SS, Minuk GY, Shapiro CM, et al. Health and socioeconomic status differences among antibody hepatitis C positive and negative transfusion recipients, 1986-1990. *Can J Public Health-Rev Can Sante Publ*. 2003 Mar-Apr;94(2):130-4.
226. Hopwood M, Treloar C. Resilient coping: applying adaptive responses to prior adversity during treatment for hepatitis C infection. *J Health Psychol*. 2008;13(1):17-27.
227. Kinder M. The lived experience of treatment for hepatitis C. *Gastroenterol Nurs*. 2009;32(6):401-8.
228. Kozanoglu E, Canataroglu A, Abayli B, Colakoglu S, Goncu K. Fibromyalgia syndrome in patients with hepatitis C infection. *Rheumatol Int*. 2003 Sep;23(5):248-51.
229. Kraus MR, Schafer A, Csef H, Scheurlen M, Faller H. Emotional state, coping styles, and somatic variables in patients with chronic hepatitis C. *Psychosomatics*. 2000 Sep-Oct;41(5):377-84.
230. Lang CA, Conrad S, Garrett L, Battistutta D, Cooksley WGE, Dunne MP, et al. Symptom

prevalence and clustering of symptoms in people living with chronic hepatitis C infection. *J Pain Symptom Manage*. 2006 Apr;31(4):335-44.
231. Lehman CL, Cheung RC. Depression, anxiety, post-traumatic stress, and alcohol-related problems among veterans with chronic hepatitis C. *Am J Gastroenterol*. 2002 Oct;97(10):2640-6.
232. McAndrews MP, Farcnik K, Carlen P, Damyanovich A, Mrkonjic M, Jones S, et al. Prevalence and significance of neurocognitive dysfunction in hepatitis C in the absence of correlated risk factors. *Hepatology*. 2005 Apr;41(4):801-8.
233. McDonald J, Jayasuriya R, Bindley P, Gonsalvez C, Gluseska S. Fatigue and psychological disorders in chronic hepatitis C. *J Gastroenterol Hepatol*. 2002 Feb;17(2):171-6.
234. McKenna O, Cunningham C, Blake C. Socio-demographic and clinical features of Irish iatrogenic hepatitis C patients: a cross-sectional survey - art. no. 323. *BMC Public Health*. 2009 Sep;9:323-.
235. Nagano J, Nagase S, Sudo N, Kubo C. Psychosocial stress, personality, and the severity of chronic hepatitis C. *Psychosomatics*. 2004 Mar-Apr;45(2):100-6.
236. Piche T, Gelsi E, Schneider SM, Hebuterne X, Giudicelli J, Ferrua B, et al. Fatigue is associated with high circulating leptin levels in chronic hepatitis C. *Gut*. 2002 Sep;51(3):434-9.
237. Poynard T, Cacoub P, Ratziu V, Myers RP, Dezailles MH, Mercadier A, et al. Fatigue in patients with chronic hepatitis C. *J Viral Hepatitis*. 2002 Jul;9(4):295-303.
238. Silberbogen AK, Janke EA, Hebenstreit C. A closer look at pain and hepatitis C: Preliminary data from a veteran population. *J Rehabil Res Dev*. 2007;44(2):231-44.
239. Weissenborn K, Krause J, Bokemeyer M, Hecker H, Schuler A, Ennen JC, et al. Hepatitis C virus infection affects the brain - evidence from psychometric studies and magnetic resonance spectroscopy. *J Hepatol*. 2004 Nov;41(5):845-51.
240. Yovtcheva SP, Rifai MA, Moles JK, Van der Linden BJ. Psychiatric comorbidity among hepatitis C-positive patients. *Psychosomatics*. 2001 Sep-Oct;42(5):411-5.
241. Castera L, Constant A, Bernard PH, de Ledinghen V, Couzigou P. Psychological impact of chronic hepatitis C: Comparison with other stressful life events and chronic diseases. *World J Gastroenterol*. 2006a Mar;12(10):1545-50.
242. Craine N, Walker M, Carnwath T, Klee H. Hepatitis C testing and injecting risk behaviour: the results of a UK based pilot study. *Int J Drug Policy*. 2004;15:115-22.
243. Gill ML, Atiq M, Sattar S, Khokhar N. Psychological implications of hepatitis C virus diagnosis. *J Gastroenterol Hepatol*. 2005 Nov;20(11):1741-4.
244. Harris M. Troubling biographical disruption: narratives of unconcern about hepatitis C diagnosis. *Sociol Health Illn*. 2009 Nov;31(7):1028-42.
245. Sutton R, Treloar C. Chronic illness experiences, clinical markers and living with hepatitis C. *J Health Psychol*. 2007 Mar;12(2):330-40.
246. Tompkins CN, Wright NM, Jones L. Impact of a positive hepatitis C diagnosis on homeless injecting drug users: a qualitative study. *Br J Gen Pract*. 2005 Apr;55(513):263-8.
247. Crockett B, Gifford SM. "Eyes Wide Shut": narratives of women living with hepatitis C in Australia. *Women Health*. 2004;39(4):117-37.
248. Fraser S, Treloar C. 'Spoiled identity' in hepatitis C infection: the binary logic of despair. *Crit Public Health*. 2006;16(2):99-110.
249. Golden J, Conroy RM, O'Dwyer AM, Golden D, Hardouin JB. Illness-related stigma, mood and adjustment to illness in persons with hepatitis C. *Soc Sci Med*. 2006 Dec;63(12):3188-98.
250. Hopwood M, Treloar C, Bryant J. Hepatitis C and injecting-related discrimination in New South Wales, Australia. *Drug-Educ Prev Policy*. 2006 Feb;13(1):61-75.
251. Zickmund S, Ho EY, Masuda M, Ippolito L, LaBrecque DR. "They treated me like a leper". Stigmatization and the quality of life of patients with hepatitis C. *J Gen Intern Med*. 2003 Oct;18(10):835-44.

252. Banwell C, Bammer G, Gifford SM, O'Brien ML. Australian lesbian and bisexual women's health and social experiences of living with hepatitis C. *Health Care Women Int*. 2005 Apr;26(4):340-54.
253. Braitstein P, Li K, Kerr T, Montaner JSG, Hogg RS, Wood E. Differences in access to care among injection drug users infected either with HIV and hepatitis C or hepatitis C alone. *AIDS Care*. 2006;18(7):690-3.
254. Butt G, Paterson BL, McGuinness LK. Living with the stigma of hepatitis C. *West J Nurs Res*. 2008;30(2):204-21.
255. Harris M. Living with hepatitis C: the medical encounter. *N Zealand Sociol*. 2005;20(1):4-19.
256. Paterson BL, Butt G, McGuinness L, Moffat B. The construction of hepatitis C as a chronic illness. *Clin Nurs Res*. 2006 Aug;15(3):209-24.
257. Schackman BR, Teixeira PA, Weitzman G, Mushlin AI, Jacobson IM. Quality-of-life tradeoffs for hepatitis C treatment: do patients and providers agree? *Med Decis Making*. 2008 Mar-Apr;28(2):233-42.
258. Taylor-Young P, Hildebrandt E. The multidimensional burden of hepatitis C and its treatment: a case study. *Gastroenterol Nurs*. 2009 May-Jun;32(3):180-7.
259. Zickmund S, Hillis SL, Barnett MJ, Ippolito L, LaBrecque DR. Hepatitis C virus-infected patients report communication problems with physicians. *Hepatology*. 2004 Apr;39(4):999-1007.
260. Khokhar OS, Lewis JH. Reasons why patients infected with chronic hepatitis C virus choose to defer treatment: do they alter their decision with time? *Dig Dis Sci*. 2007 May;52(5):1168-76.
261. McNally S, Temple-Smith M, Sievert W, Pitts MK. Now, later or never? Challenges associated with hepatitis treatment. *Aust N Z J Public Health*. 2006;30(5):422-7.
262. Ogawa LM, Bova C. HCV treatment decision-making substance use experiences and hepatitis C treatment decision-making among HIV/HCV Coinfected Adults. *Subst Use Misuse*. 2009;44(7):915-33.
263. Rowan PJ, Dunn NJ, El-Serag HB, Kunik ME. Views of hepatitis C virus patients delayed from treatment for psychiatric reasons. *J Viral Hepat*. 2007 Dec;14(12):883-9.
264. Treloar C, Hopwood M. "Look, I'm fit, I'm positive and I'll be all right, thank you very much": coping with hepatitis C treatment and unrealistic optimism. *Psychol Health Med*. 2008 May;13(3):360-6.
